# The Chaotic Terrains of Mercury Reveal a History of Planetary Volatile Retention and Loss in the Innermost Solar System

**DOI:** 10.1038/s41598-020-59885-5

**Published:** 2020-03-16

**Authors:** J. Alexis P. Rodriguez, Gregory J. Leonard, Jeffrey S. Kargel, Deborah Domingue, Daniel C. Berman, Maria Banks, Mario Zarroca, Rogelio Linares, Simone Marchi, Victor R. Baker, Kevin D. Webster, Mark Sykes

**Affiliations:** 1 Planetary Science Institute, 1700 E Fort Lowell Road, Suite 106, Tucson, AZ USA; 2 Department of Planetary Sciences, University of Arizona, Tucson, AZ USA; 3 NASA Goddard Space Flight Center, Greenbelt, MD USA; 4 External Geodynamics and Hydrogeology Group, Department of Geology, Autonomous University of Barcelona, 08193 Bellaterra, Barcelona Spain; 5 Southwest Research Institute, 1050 Walnut St, Suite 300, Boulder, CO USA; 6 Department of Hydrology and Atmospheric Sciences, University of Arizona, Tucson, AZ USA

**Keywords:** Geomorphology, Inner planets

## Abstract

Mercury’s images obtained by the 1974 Mariner 10 flybys show extensive cratered landscapes degraded into vast knob fields, known as chaotic terrain (AKA hilly and lineated terrain). For nearly half a century, it was considered that these terrains formed due to catastrophic quakes and ejecta fallout produced by the antipodal Caloris basin impact. Here, we present the terrains’ first geologic examination based on higher spatial resolution MESSENGER (MErcury Surface Space ENvironment GEochemistry and Ranging) imagery and laser altimeter topography. Our surface age determinations indicate that their development persisted until ~1.8 Ga, or ~2 Gyrs after the Caloris basin formed. Furthermore, we identified multiple chaotic terrains with no antipodal impact basins; hence a new geological explanation is needed. Our examination of the Caloris basin’s antipodal chaotic terrain reveals multi-kilometer surface elevation losses and widespread landform retention, indicating an origin due to major, gradual collapse of a volatile-rich layer. Crater interior plains, possibly lavas, share the chaotic terrains’ age, suggesting a development associated with a geothermal disturbance above intrusive magma bodies, which best explains their regionality and the enormity of the apparent volume losses involved in their development. Furthermore, evidence of localized, surficial collapse, might reflect a complementary, and perhaps longer lasting, devolatilization history by solar heating.

## Introduction

The finding of numerous volatile-bearing surfaces^1–7^ on Mercury comprises one of the most critical discoveries by the MESSENGER (MErcury Surface Space ENvironment GEochemistry and Ranging) spacecraft. The measured global high abundances of S^1^, Cl^2^, and K^5^ reveal a significant surface record of volatile-bearing materials. Also, local polar H_2_O ice form deposits within numerous permanently shadowed crater interiors in the planet’s polar regions^8–10^. Currently documented evidence of surface modifications due to the removal of  volatiles includes Mercury’s hollows, which are shallow, flat-floored, irregular, rimless depressions with bright interiors, and halos^11–14^. These features are relatively small, exhibiting an average depth of 24 ± 16 m^13^. Therefore, their  origin is thought to represent minor mass losses produced by the local sublimation of near-surface geologic material that included large volumes of volatiles (i.e., volatile-rich compounds)^11–14^. However, these observations do not constrain the total thickness, and global distribution of the planet’s upper crustal volatile-rich zones.

**Figure 1:**
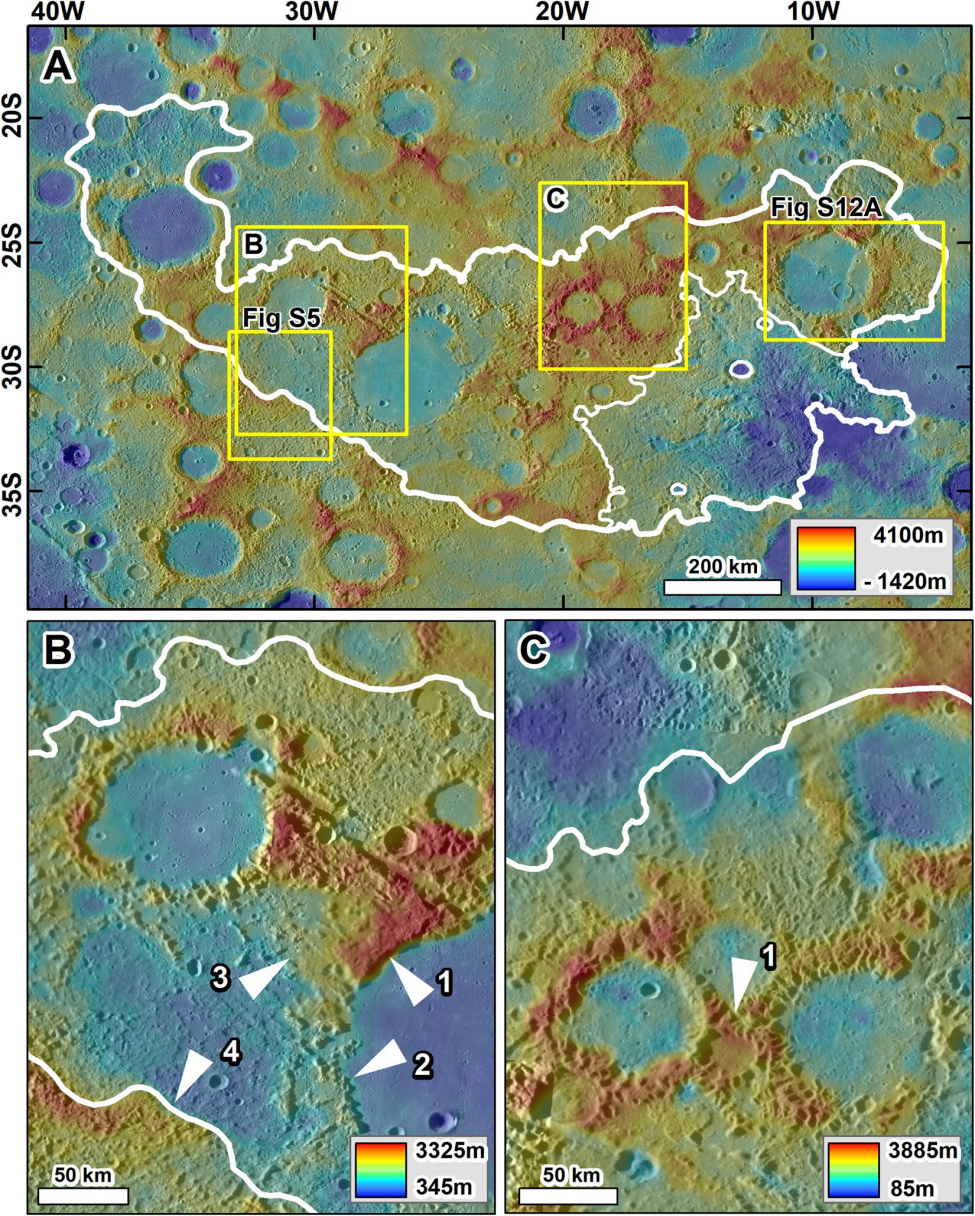
(**A**) Extent of a vast chaotic terrain (white outline) at the antipode of the Caloris basin (~5 × 10^5^ km^2^). The context and locations of panels B and C, and Figs. S5 and S12A are indicated. **(B)** Zoom in showing variable magnitudes of collapse, which includes a relatively unmodified rim section that is smoothed but not broken into knobs (arrow 1). This area adjoins another part of the rim that has been almost entirely removed (arrow 2). The adjacent intercrater regions also exhibit deep and abrupt relief losses (arrows 3 & 4). **(C)** Zoom in showing a cluster of three impact craters with diameters ranging from ~ 25 km to ~75 km. Note that while the rims exhibit abrupt variations in their collapse magnitudes (arrow 1), the distribution of the knobby materials retains the craters’ circularity. All panels are parts of a MESSENGER Mercury global DEM (~665 m/px; credits to^46–48^): draped over MESSENGER Mercury Dual Imaging System (MDIS) global base map (~166 m/px; credits to^47,49,50^).

The hollows are the smallest described surface modifications due to endogenic processes thus far detected on Mercury. At the other end of the spectrum, as we present here, are the chaotic terrains (AKA hilly and lineated terrain^15^) that are antipodal to the Caloris basin (Fig. 1A), and which the 1974 Mariner 10 flybys first identified^16^. These terrains form the planet’s most extensive geomorphologic expression of landscape destruction and consist of immense fields of knobs (Fig. 1). Their development modified vast expanses of regional intercrater plains^17^, a geologic unit that covers about one-third of the surface area surveyed by Mariner 10^18,19^. Using MESSENGER spacecraft data, Whitten *et al*.^20^ proposed that volcanic material emplaced between pre-Tolstojan (older than ~4 Ga^21^) and the Tolstojan Period (~3.9–4.0 Ga^21,22^) likely dominates these plains. These age determinations indicate that the onset of chaotic terrain formation must have commenced after ~3.9 Ga.

Schultz and Gault^23^ suggested that seismically-induced landslides and the emplacement of ejecta fallout materials, due to the Caloris basin impact, best explains the extensive antipodal areas of chaotic terrain occurrence (Fig. 1A). They proposed that the resulting magnitude of resurfacing was so intense that only the largest of the preexisting impact craters would have remained within the modified landscapes. Some of the most investigated geologic terrains resulting from antipodal basin impacts occur on the Moon^23–27^. Outstanding possible examples of these landslides and ejecta fallout deposits exist in the lunar Mare Ingenii region (Figs. S1 and S2), which is antipodal to Mare Imbrium^23,24^, and thereby provide a comparative landscape for the Mercurian chaotic terrains. As we show in this article, the impact-generated, seismically-induced landscape character of the Mare Ingenii region contrasts considerably with the geomorphic, topographic, and structurally modified landscape features of Mercury’s Caloris-antipodal chaotic terrains.

Furthermore, the Schultz and Gault’s hypothesis implies near simultaneity in the formation of the basin and its antipodal chaotic terrain region. Hence, in this theoretical context, the basin’s age would also represent that of the chaotic terrains. There is a significant body of work that has attempted to determine the Caloris basin’s age. Crater dating of the basin’s interior and peripheral plains, as well as of its rim materials, yield ages that range between ~3.9 and ~3.7 Ga^22,28–31^. While these surface materials show similar crater size-frequency distributions^29^, the rim materials exhibit higher crater densities for any given diameter^28,30^. This discrepancy likely originates because the interior plains might have been emplaced significantly after the Caloris impact^32^. In this case, there should be craters that formed on the basin’s floor, and which the plains material subsequently partly buried. However, geomorphic evidence of such a crater burial event is lacking^32^. A further source of controversy concerns the origin of the circum-Caloris knobby plains of the Odin Formation. While its morphology and stratigraphy are consistent with Caloris basin ejecta^15,16,28^, it exhibits the lowest crater density of all the plains associated with Caloris^28^. Thus, if the crater populations are representative of age, then these materials are likely not part of the Caloris ejecta^28^. In the light of these uncertainties, Denevi *et al*.^22^ performed impact crater count statistics on the basin’s rim materials and obtained an age of ~3.8 Ga, which is the most reliable age determination thus far. As discussed in the next section, however, our age determinations for surface modification of the antipodal chaotic terrains are widely disparate to the formation age of the Caloris basin. Additionally, numerical simulations suggest that the Caloris impact would have created an area of surface disturbance smaller than the basin’s antipodal chaotic terrains^33^. Therefore, the development of the antipodal chaotic terrain requires an explanation largely disssociated with the Caloris impact.

Here, we present the first detailed morphologic investigation of these chaotic terrains using MESSENGER datasets. Our results support an origin due to the widespread, yet non-catastrophic, surface elevation losses (hereon, referred to as collapse) of a multi-kilometer thick upper crustal volatile-rich layer.

## Results

### Emergence of a new hypothesis: post-Caloris chaotic terrain development

Here, we document crucial MESSENGER data-based results revealing inconsistencies regarding the hypothesis that the Caloris impact produced its antipodal chaotic terrain (see Supplementary Materials for Synthesis of Mapping Approach and Methodology for Age Determinations Based on Crater Count Statistics).

#### The age disparity problem

**Figure 2:**
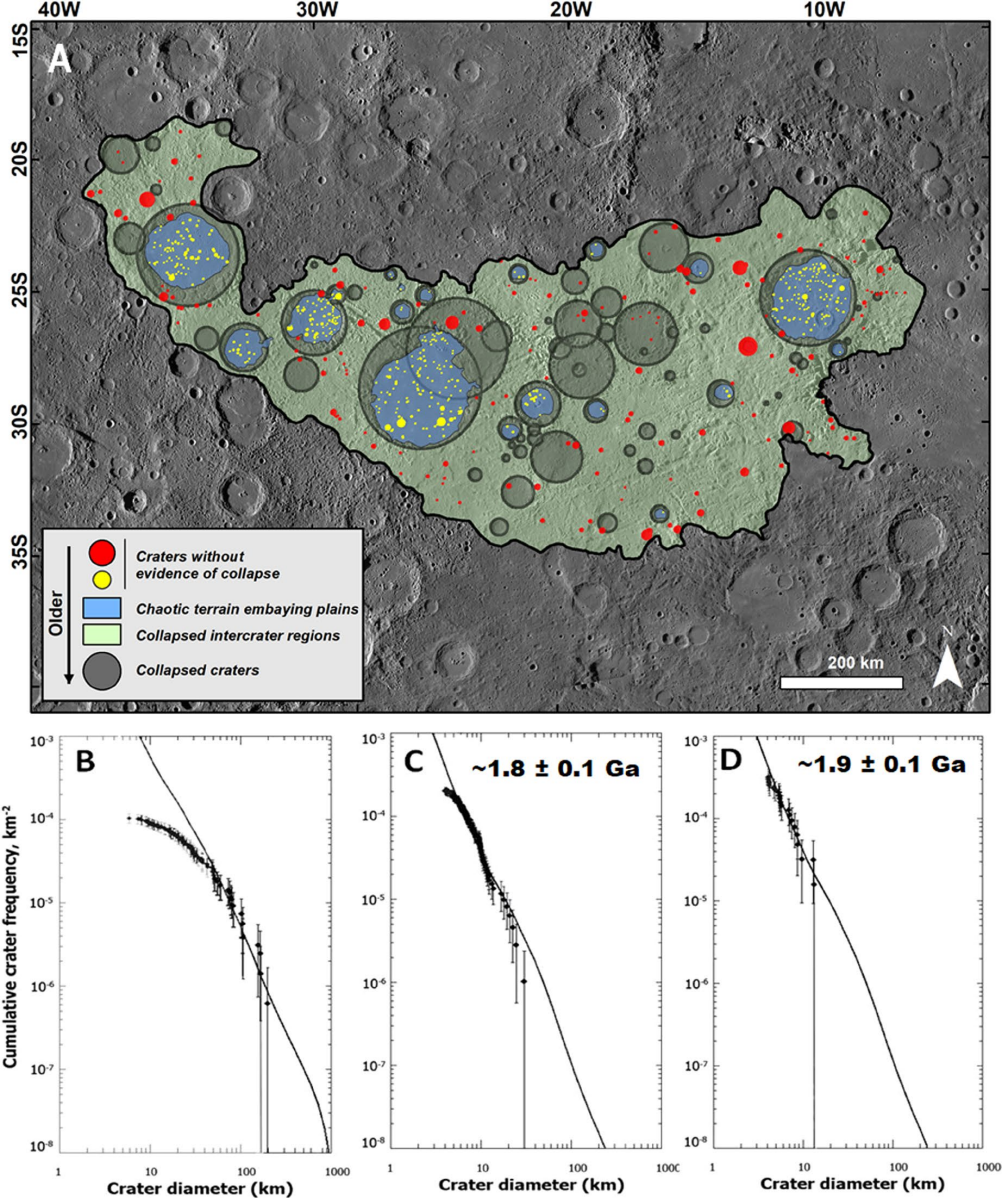
(**A**) Chaotic terrain located at and near the Caloris basin’s antipode, and the distribution of impact craters that were used to generate the plots in panels B–D. To generate these plots we counted all visible craters ≥5 km in diameter. **(B)** Crater size-frequency distribution (SFD) for the population of collapsed craters (dark circles) superposing the entire chaotic terrain region (fit to the Main Belt Asteroid (MBA) production for craters ≥50 km in diameter). Note a depletion in collapsed craters smaller than ~50 km. The rollover is not the result of insufficient resolution because it is not reflected in the SFD of the craters with no evidence of collapse that occur in the same chaotic terrain region. **(C)** Modeled age (using the Near-Earth Object–NEO–production function and craters ≥5 km in diameter) derived from a population of craters that shows no evidence of collapse (red circles), and which superposes the chaotic terrain’s areas affected by collapse. **(D)** Modeled age (using the NEO production function and craters ≥5 km in diameter) from a population of craters that shows no evidence of collapse (yellow circles), and which superposes the chaotic terrain’s crater interior plains. Note that these plains, probably lavas, were emplaced following the main phase of chaotic terrain formation as indicated by the fact that their margins embay (and thus postdate) the chaotic terrain’s knobs and other collapsed features. The base image in panel (A) is part of a MESSENGER Mercury Dual Imaging System (MDIS) global base map (~166 m/px; credits to^47,49,50^).

If the chaotic terrains had formed due to the Caloris impact ~3.8 Ga, any newly formed craters would have been fresh and occurred with a production crater-size-frequency distribution age equal to that of Caloris. The size-frequency distribution of collapsed craters in the chaotic terrain antipodal to the Caloris basin reveals a substantial depletion of craters < ~50 km in diameter and a nearly complete loss of craters <10 km (Fig. 2A,B). The curve goes almost horizontal at <10 km sizes, implying that nearly all such small craters were completely destroyed, and craters larger than ~50 km were preserved but degraded. These observations indicate that the collapse leading to chaotic terrain formation was sustained and hence also reduced the retention potential of impact craters. Our counts of impact craters unmodified by collapse indicate that collapse mostly ceased ~1.8 +/− 0.1 Ga (Fig. 2A,C), which is ~2 Ga after the Caloris basin formation event. However, the onset and duration of the collapse remain uncertain.

#### Evidence of pervasive collapse within the chaotic terrain

**Figure 3:**
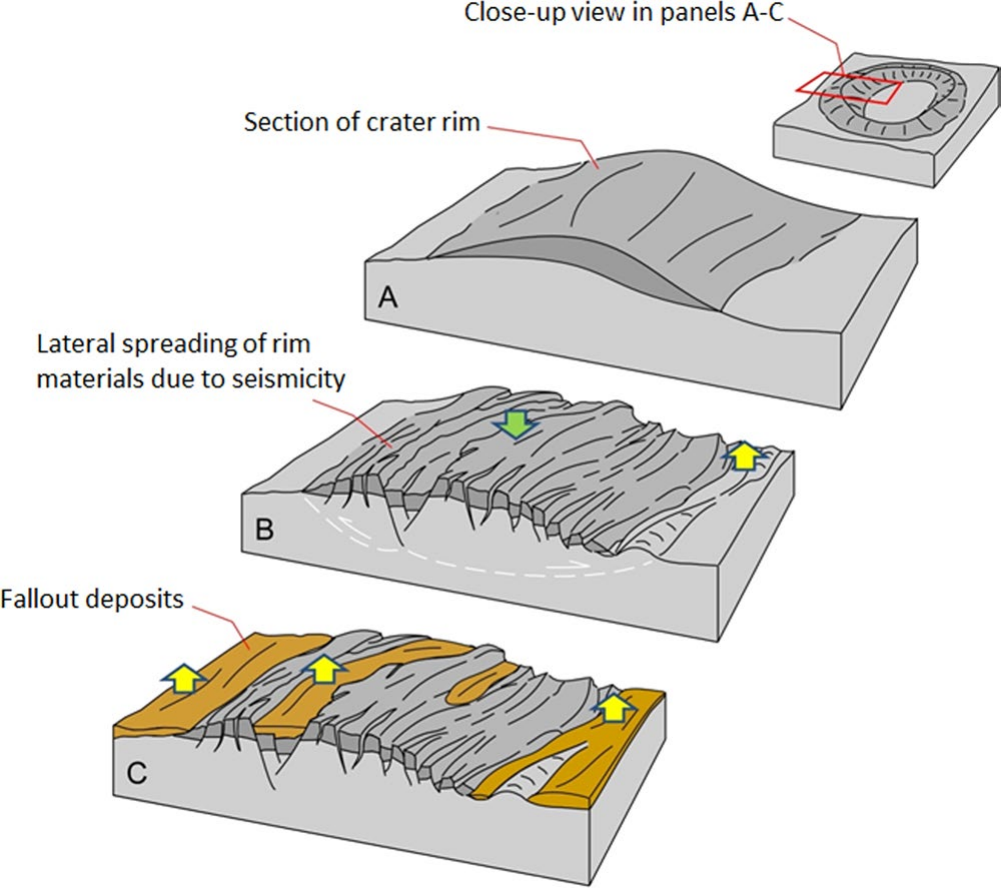
(**A**) Perspective cross-sectional view of part of an impact crater rim. **(B)** Seismic activity reduces the mean elevation of the crater rim (green arrow). However, it increases the mean elevation of adjoining plains as the rim-forming materials collapse outward (yellow arrow). **(C)** The superposition of ejecta fallout materials over these terrains, which could have increased the region’s mean surface elevation. See Figs. S1 and S2 for the case study region on the Moon.

We have identified landscape features within the lunar Mare Ingenii region that are in agreement with the hypothesis that a major antipodal impact in the Mare Imbrium produced powerful regional moonquakes as well as massive fallout deposition and scouring. These include large landslide runouts–probably seismically induced–shed by crater rims (Fig. S1). We also observe possible ejecta fallout such as lineated materials, which partly cover crater rims and form intercrater deposits with elevations close to those of nearby crater rims (Fig. S2). We note that while these disturbed landscapes would have had reduced reliefs, including raised inter/intra-crater regions due to sedimentation and ridges lowered by collapse, the regions’ mean surface elevation would have remained unchanged or increased in zones significantly affected by ejecta fallout emplacement (Fig. 3).

**Figure 4:**
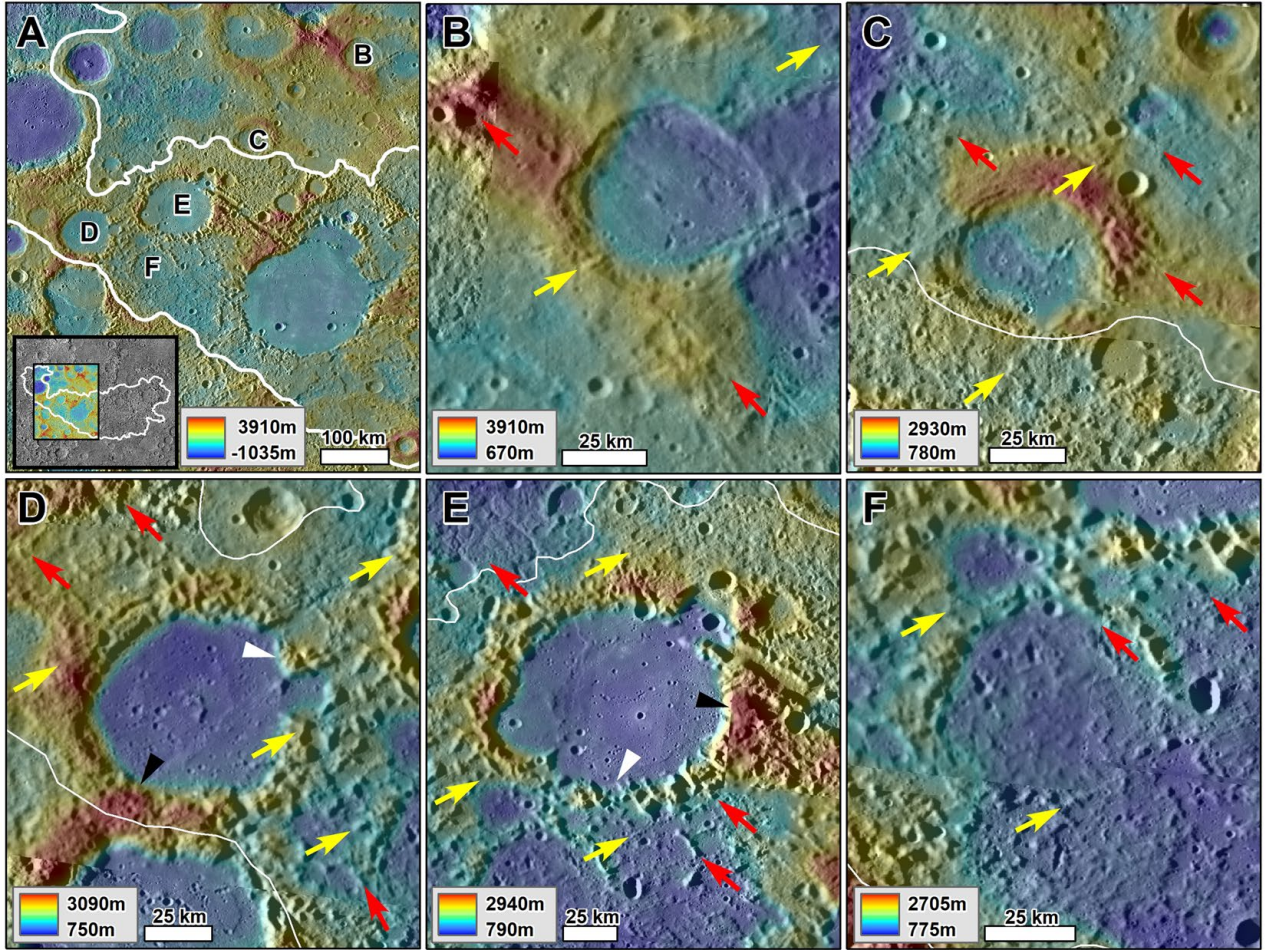
(**A**) Context and locations for panels B-F, which show impact craters of similar sizes but with varying degrees of modification into chaotic terrains. **(B,C)** Examples of regions affected by “incipient collapse”. Note the presence of craters affected by possible extensional faulting (lineations), but not by significant collapse. **(D–F)** Examples of the “prominent collapse” of crater rims and intercrater regions degraded into clusters of immense knobs. **(E)** Example of “extreme collapse” areas where there has been almost complete removal of the pre-collapse landscape and knob fields. Note that each morphologic zone retains the regional structural NW and NE trends (red and yellow arrows respectively). The black and white triangles identify the best preserved and deepest collapsed rim areas, respectively. All panels are parts of a MESSENGER Mercury global DEM (~665 m/px; credits to^46–48^) draped over MESSENGER Mercury Dual Imaging System (MDIS) global base map (~166 m/px; credits to^47,49,50^).

**Figure 5:**
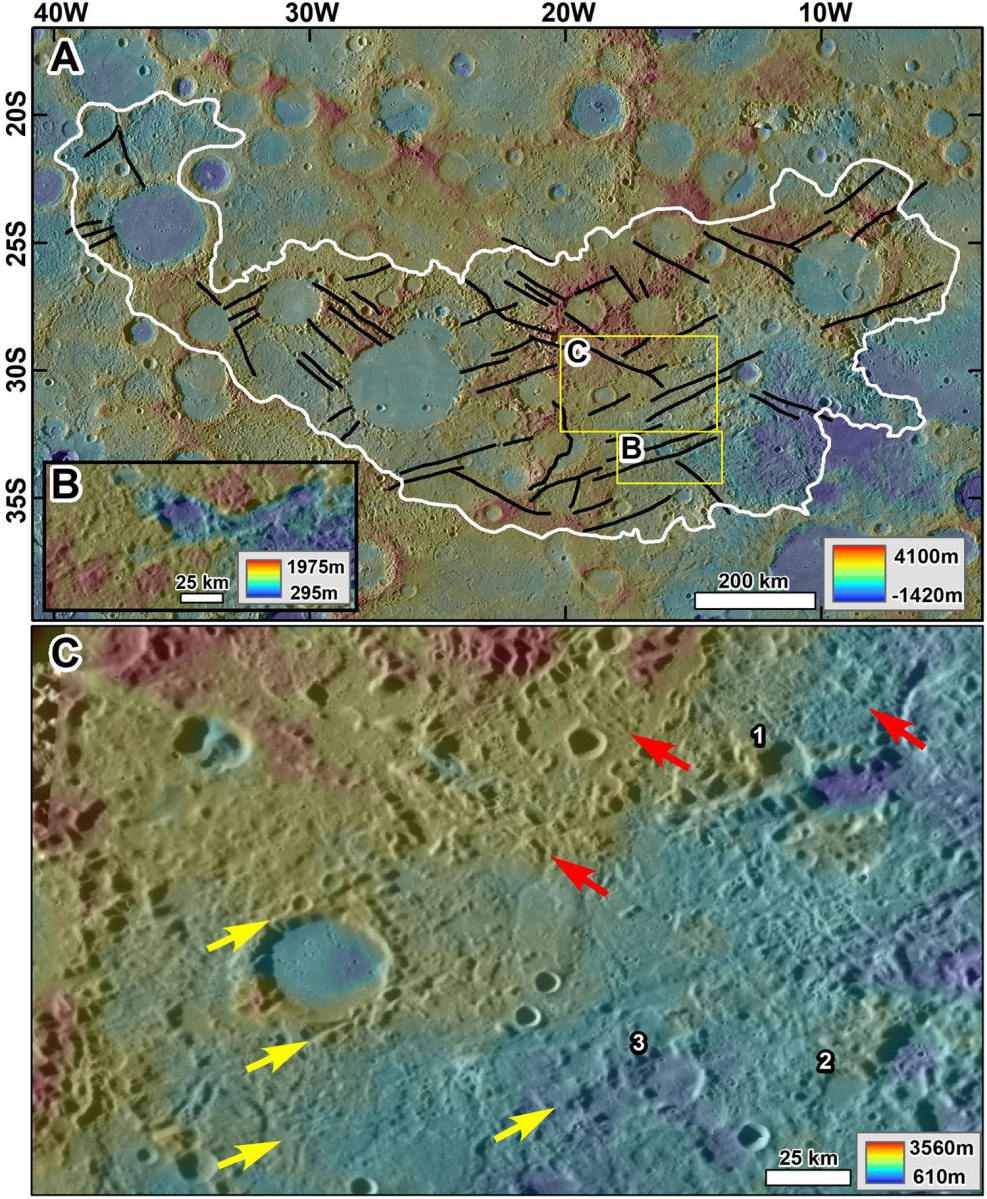
(**A**) Map of the largest groove systems in the chaotic terrain antipodal to the Caloris basin. The structures have prevailing NW and NE trends. **(B)** Close-up view on one of the grooves, which has a width of ~20 km. Note that the groove’s relief is due to collapse. **(C)** Zoom in showing a chaotic terrain area that includes the remnants of smaller and more closely spaced grooves (yellow and red arrows, respectively, identify dominant NE and NW trends). The bottom-center yellow arrow is aligned to the head of a large groove. Numbers 1–3 indicate the locations of relatively small craters (~20 km in diameter) that underwent collapse. All panels are parts of a MESSENGER Mercury global DEM (~665 m/px; credits to^46–49^) draped over MESSENGER Mercury Dual Imaging System (MDIS) global base map (~166 m/px; credits to^47,49,50^).

The Caloris antipodal chaotic terrain lack the types of crater rim landslide and ejecta fallout morphologies that are present in the disturbed terrains of the Imbrium antipode terrain of Mare Ingenii. Instead, their landscapes are characterized by broad dissection patterns, in which individual grooves breach the entire relief of crater rims and deeply cut into intercrater regions (e.g., Figs. 1B,C, 4 and 5). These grooves have widths ranging from a few kilometers to ~20 km and consistently exhibit NW and NE orientations (e.g., Figs. 1B,C, 4C,D and 5C). The breached craters have diameters ranging from ~20 km to ~200 km, and their rim gaps generally align to the orientations of the intercrater regions’ grooves (e.g., Figs. 1B,C, 4C,D and 5C). These observations are consistent with an origin due to collapse throughout both elevated rim areas and lower-lying plains.

#### Evidence of non-catastrophic chaotic terrain development

The catastrophic formation of the chaotic terrains by massive quakes and ejecta fallout emplacement would have most likely destroyed all but the largest of the preexisting cratered landscapes. However, we have identified the retention of the pervasive linear patterns noted above – a possible underlying structural fabric - and the circularity of craters with diameters down to ~20 km across (Figs. 1C, 4 and 5). These observations indicate that the collapse did not involve significant horizontal ground shifting, suggesting gradual (non-catastrophic) and structurally controlled *in-situ* development.

### Paleogeographic reconstructions and volume loss estimates of the Caloris antipodal chaotic terrain

#### Morphologic mapping and categories of collapsed terrains

Our investigation reveals evidence of varying magnitudes of collapse within Mercury’s chaotic terrains (Figs. 1B, 4). The chaotic terrains’ NE and NW-trending linear patterns, preserved in the regional alignment of knobs and grooves, locally coexist with the arcuate patterns of knobs present in collapsed crater rims (e.g., Figs. 1, 4). The linear patterns are traceable over hundreds of kilometers of cratered terrains and intercrater regions (Fig. 5A), suggesting that the collapse was structurally controlled. The grooves include large troughs (e.g., Fig. 5B) as well as narrow, closely spaced valleys (Fig. 5C). These dissection patterns are distinct morphologically from secondary crater clusters, which typically form systems of pit chains. However, it appears that impact ejecta fallout might have excavated some large grooves (e.g., Fig. 5B).

**Figure 6:**
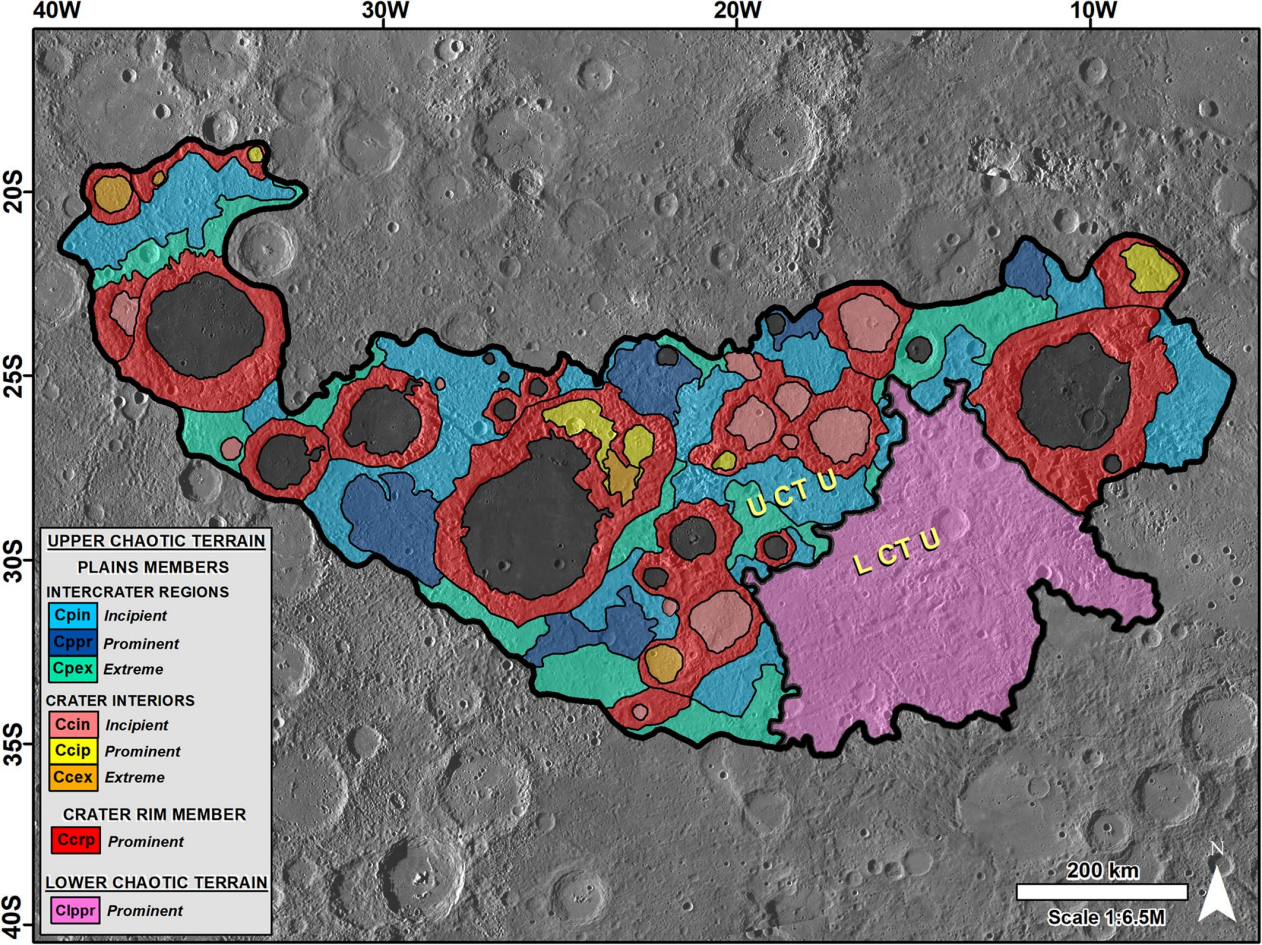
Map showing the various types of terrain morphologic zones at and near the Caloris basin’s antipode. We mapped seven distinct members within the UCTU (see Table 1 below for their descriptions, and Fig. S3 for their statistical morphometric characterizations) and the LCTU as a single unit. Dark crater interiors are probable lava plains that we dated as ~1.9 Ga in Fig. 2. The base image is part of a MESSENGER Mercury Dual Imaging System (MDIS) global base map (~166 m/px; credits to^47,49,50^).

We mapped two geomorphologic units; the Upper Chaotic Terrain Unit (UCTU) (~3.9 x10^5^ km^2^), and the Lower Chaotic Terrain Unit (LCTU) (~1 x10^5^ km^2^) (Fig. 6). The presence of transitional terrains, many of which are separated by abrupt topographic and morphologic boundaries, indicates that the magnitude of resurfacing associated with the generation of these terrains was regionally variable (e.g., Fig. 4). We subdivided the UCTU into various morphologic zones, which we mapped as distinct members. These include the cratered terrains (1) marked by extensive systems of linear grooves; (2) in which the surfaces adjoining the grooves are degraded into fields of closely spaced knobs; and (3) in which broad plains separate the knobs that were produced by the rupturing of the pre-existing landscapes. We refer to these members as incipient, prominent, and extreme zones of chaotic terrain modification, respectively. Each member exhibits distinct surface relief characteristics, in which the “prominent” collapse member has the largest relief ranges (Fig. S3).

Based on these morphologic zone categories, we mapped seven UCTU members, including three plains members and three crater interior members, which distinguish incipient, prominent, and extreme gradational modifications; and a crater rim member characterized by prominent modifications (Fig. 6; Table 1). We did not subdivide the LCTU [type area centered at 30° 48′ S, 15° 10′ W]. This unit is characterized by a lack of large impact craters (perhaps due to a higher magnitude of resurfacing), and the fact that it includes immense knobs that generally cluster along regional grooves (Fig. S4). The unit occupies the lowest chaotic terrain areas and a break-in-slope at the 1,800 m elevation defines most of the unit’s contact with the UCTU. The UCTU’s mean elevation is ~1840 m, while the LCTU’s is ~850 m, or ~1000 m lower.

**Table 1:** Description of Upper Chaotic Terrain Unit (UCTU) members.

PLAINS MEMBERS
INTERCRATER REGIONS
**Chaotic Plains Incipient** [Cpin; Type area centered at 34° 00′ S, 24° 20′ W]: Plains, mostly situated within intercrater regions, which include widespread occurrences of low-relief knobs and shallow NW and NE-trending grooves.
**Chaotic Plains Prominent** [Cppr; Type area centered at 27° 00′ S, 28° 07′ W]: Plains, mostly situated within intercrater regions, which include clustered occurrences of prominent knobs and ridges. These features consistently trend to the NW and NE and are locally flanked or divided by similarly oriented grooves.
**Chaotic Plains Extreme** [Cpex; Type area centered at 30° 50′ S, 30° 50′ W]: Plains, mostly situated within intercrater regions, which consist of broad low-lying areas that retain faint groove markings, and contain low-density occurrences of scattered knobs and ridges; both typically having lower relief than similar Cppr features.
CRATER INTERIORS
**Chaotic Crater Interior Incipient** [Ccin; Type area centered at 27° 40′ S, 19° 30′ W]: Crater floors that include widespread occurrences of low-relief knobs and shallow NW and NE-trending grooves.
**Chaotic Crater Interior Prominent** [Ccip; Type area centered at 27° 45′ S, 24° 10′ W]: Crater floors consisting of extensive occurrences of topographically prominent, clustered knobs and ridges with lengths that consistently trend to the NW and NW. These features are locally flanked or divided by similarly oriented grooves.
**Chaotic Crater Interior Extreme** [Ccex; Type area centered at 29° 00′ S, 23° 28′ W]: Crater floors consisting of broad low-lying areas that retain faint groove markings, which separate scattered knobs and ridges.
CRATER RIM MEMBER
**Chaotic Crater Rim Prominent** [Ccrp; Type area centered at 28° 20′ S, 30° 24′ W]: Extensively breached crater rims (~ >50 km diameter) in which the breached sections align to the NW and NW regional trends. These topographic gaps separate high-relief knobs and ridges. Smooth plains, which are probably lavas, typically occupy the interior of these craters and embay the knobs and ridges.

Note that each of these members’ descriptions is typical of their extents. However, their margins and some interior areas present variability due to the transitional nature of their contacts.

#### Estimates of volume losses due to chaotic terrain development

**Figure 7:**
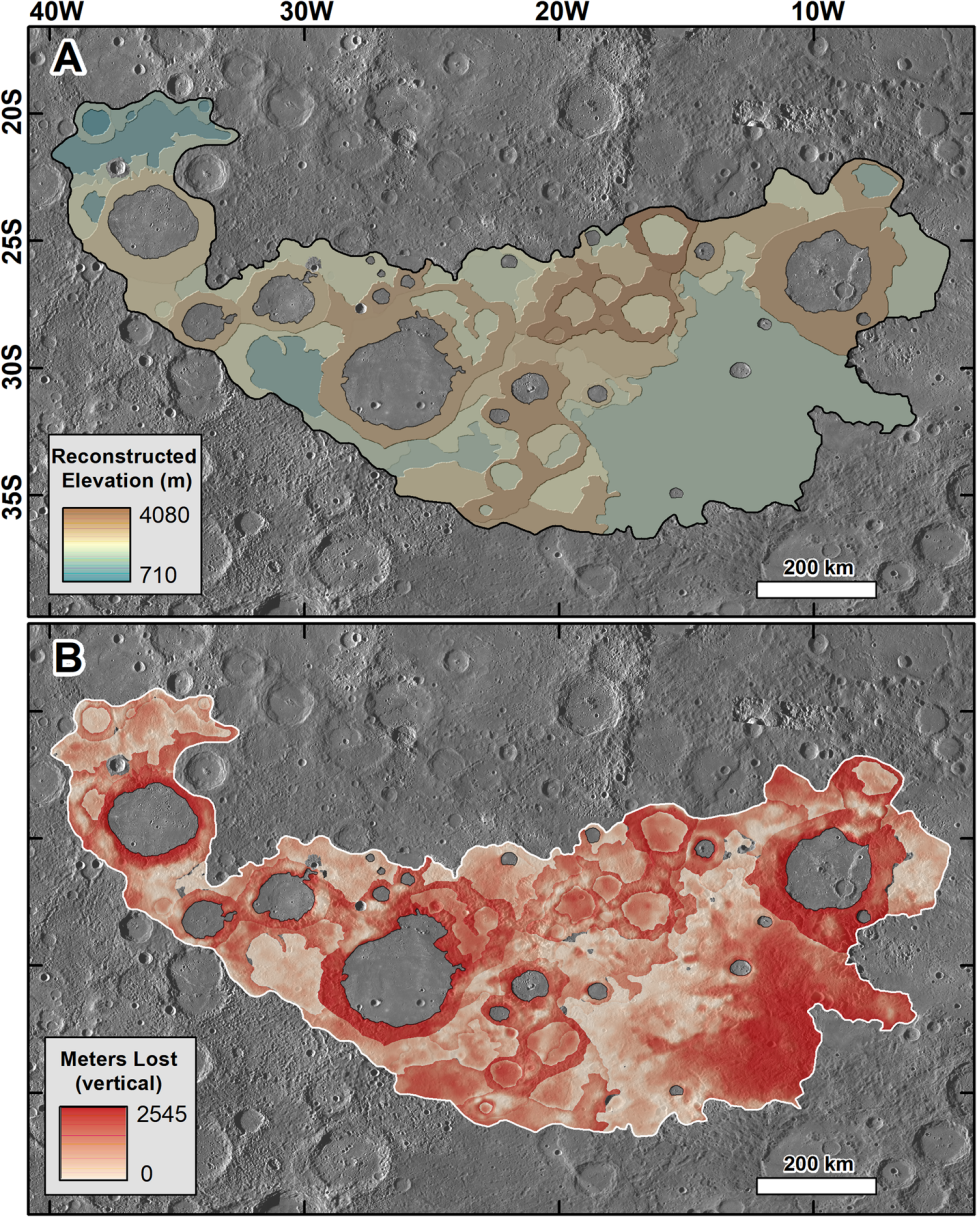
(**A**) Digital elevation model showing a reconstruction of the pre-collapse landscapes in the Caloris basin antipode, which was produced using topographic extrapolations from the least degraded landscape elements within each of the areas mapped in Fig. 6. **(B)** Digital elevation model showing the estimated relief losses produced during the collapse, which we produced by subtracting the current from the reconstructed topography. The base images are parts of a MESSENGER Mercury Dual Imaging System (MDIS) global base map (~166 m/px; credits to^47,49,50^).

In our morphologic map, each incipient, prominent, and extreme zone of chaotic terrain is attributed with an origin category (i.e., intercrater plains, crater interior plains, or crater rim materials, Fig. 6). We used this classification scheme to reconstruct the landscape topography preceding chaotic terrain formation (Fig. 7A). To maximize accuracy, we reconstructed the topography of the pre-chaotic terrain landscapes  for each of the plains or crater region occurrences, each delineated by individual polygons in our geomorphic map (Fig. 6). For collapsed plains regions, our reconstructions were based on extrapolated elevations from adjoining plains that show no or lesser evidence of collapse. Even if not evident in the DEM, these reference areas might have also experienced some collapse, and so some of the  reconstructions might be topographic underestimates, potentially  including some surfaces lower than pre-collapse landscapes. On the other hand, in the reconstructions of collapse in impact crater rims we extrapolated the maximum elevation in the residual rim materials to the entire rim areas. Crater rims can exhibit variable relief, and the lowest rim sections’ reconstructions might include topographic overestimates. However, many of these rim sections adjoin collapsed plains (e.g., Fig. 1B), supporting the assumption  in our estimates that collapse formed many of the low rim areas within the mapped regions.

Subtracting the current topography from the reconstructed pre-chaotic terrain surface elevations indicates the removal of at least ~3.6 × 10^5^ km^3^ of upper crustal materials (Fig. 7B), reflecting hundreds of meters to a few kilometers of collapse resulting  from chaotic terrain development (Figs. S3 and S4). The implied immense magnitude of collapse is consistent with the observation that chaotic terrain development appears to have almost entirely destroyed craters < 10 km (Fig. 2B).

### A new model: the non-catastrophic collapse of a volatile-rich crust on Mercury

**Figure 8:**
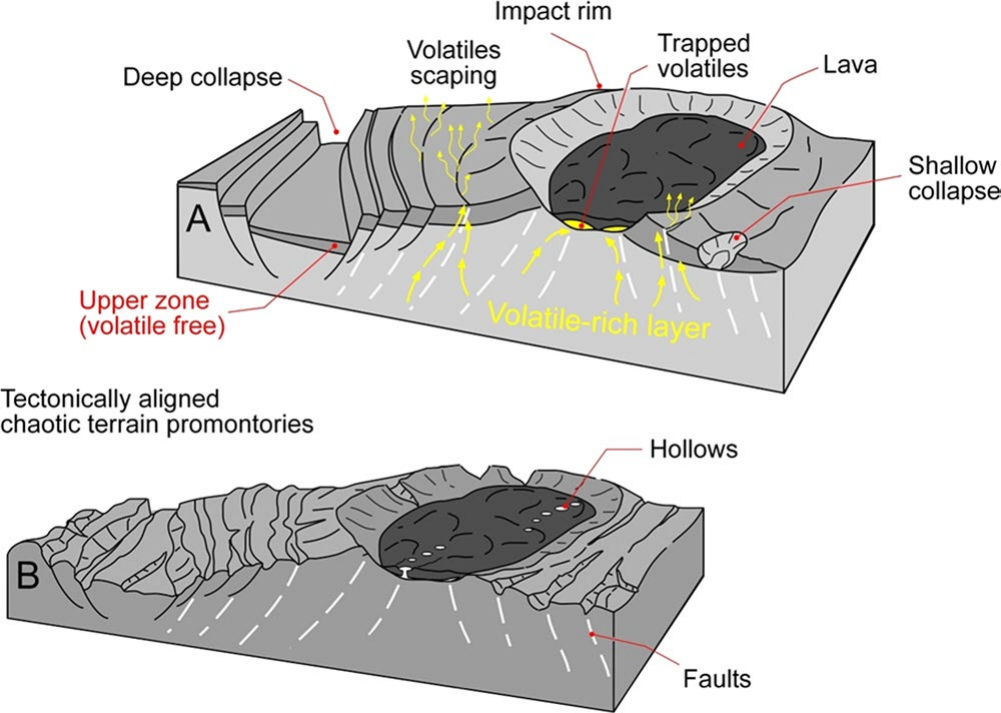
(**A**) Sketch showing a perspective cross-sectional view of Mercury’s cratered terrains undergoing structurally controlled upper crustal devolatilization and collapse. The yellow arrows trace volatile escape routes in extensive fault systems. We note the presence of an upper zone, consisting largely of lags,  generated during the devolatilization The trapping of some of the volatiles beneath impact crater interior plains could have set the conditions for the later regional hollow development. **(B)** Chaotic terrain landscape following the large-scale volatile losses from the upper crust. The illustration shows how the structurally controlled collapse produced breached rims and aligned knobs observed throughout the ancient crater rims and intercrater regions, as well as aligned hollows within the crater’s interior plains.

We attribute the immense volume losses, which we infer to have occurred during chaotic terrain formation (Fig. 7B), to widespread collapse associated with the devolatilization of hundreds of meters to a few kilometers of upper crustal materials (Fig. 8). In the context of this hypothesis, we define “collapse” as encompassing elevation losses due to (1) mass wasting associated with the sublimation of surface/near-surface volatiles and (2) gravity-driven terrain disintegration over zones of deep volatile evacuation.

#### Depth estimates of the volatile-rich crust

The maximum depth of collapse, which ranges from ~2 to ~2.5 km, occurs in association with high relief crater rims and some broad grooves (Figs. 1B, 5A,B, 7B, S3 and S4). This value range is an upper approximation to the thickness of the volatile-rich crustal zone, which underwent collapse to form the chaotic terrains. There is no evidence of collapse or subsidence into evacuated chambers or conduits (e.g., surface depressions flanked by extensional faults). This is true even in areas where the landscape has been almost completely erased due to collapse. This observation indicates that collapse took place within (vs. over) crustal materials that included large volumes of volatiles, implying that the volatile-rich crust extended to the near-surface. Further evidence of near-surface volatile-bearing materials within the chaotic terrain includes the zonal collapse of rare surface occurrences that include numerous preserved craters ≤10 km in diameter (Fig. S5). The partial rim collapse in these small craters is consistent with a near-surface volatile-rich composition and highlights a probable role of direct surface sublimation driven, or constrained, by solar luminosity. Another relevant observation is that the areas of extreme collapse (Fig. 6) consist of low-lying plains with scattered knobs that have considerably lower relief ranges than those that occur within areas of prominent collapse (e.g., Figs. 4F, S3B,C). The significance of this finding is that the knobby terrains produced during the collapse of the cratered landscapes experienced further disintegration. It also suggests that the volatile-rich materials existed within, and immediately below, the relief of the preexisting landscape.

#### A potential link to extensional tectonism

While the lineated patterns consistently exhibit NW and NE trends, they appear most pronounced in chaotic terrain zones that include knobby terrain (Fig. 4). At widespread locations, there are grooved surfaces that adjoin knobby terrains. In these regions, the knobs have lengths that typically align to the grooves’ orientations (Fig. 4D). We interpret these linear patterns preserved in the distribution of the knobs (Figs. 4, 5A) as reflecting a history of enhanced collapse over extensional faults. This interpretation is also consistent with the grooves’ parallel orientations and linearity that persists independently of topography, and which are also present at multiple scales (Figs. 5C; S6). This pattern of collapse most likely resulted as the extensional faults provided crustal pathways for the migration and escape of deeply buried volatiles (Fig. 8). Within our interpretational context, these knobs represent the remnants of fault-bounded upper crustal materials following enhanced, structurally controlled, volatile-driven collapse along the proposed extensional faults. The overall retention of the tectonic structural patterns and crater rim outlines are consistent with the degassing due to devolatilization being slow and not leading to catastrophic collapse.

#### Evidence of gradual chaotic terrain development

Our mapping outlines two distinct styles of collapse that occurred during chaotic terrain development; (1) the collapse of extensive cratered landscapes into vast fields of knobs (e.g., Fig. 4D, areas mapped as prominent collapse in Fig. 6), and (2) the collapse of many of these knobs to form low-lying plains that include widely scattered knobs (e.g., Fig. 4F areas mapped as extreme collapse in Fig. 6). These two stages are also broadly reflected in our statistical topographic characterizations of the mapped chaotic terrain morphologic zones (Figs. S3 and S4). To test whether the landscape progressively disintegrated, we examined the geomorphology of one of these low-lying plains using a MESSENGER’s Narrow-Angle Camera (NAC) Messenger Regional Targeted Mosaic (RTM). The region includes a section of a crater rim almost entirely removed by collapse as well as adjoining intercrater areas (Fig. S6A).

The high-resolution view reveals a landscape within and near the area of rim removal, which includes a smooth, low albedo surface in which underlying higher albedo materials are exposed at zones of impacts. This surface adjoins a lower-lying, topographically rougher region that also shows a higher albedo. This terrain transtion suggests the localized removal of the materials comprising the low albedo surface. The bright, lowered surface includes small hills (some <1 km across at their base), which locally retain a NW structural pattern in the form of grooves, some also with sub-kilometer widths (Fig. S6B). Our interpretation is that this terrain developed during a phase of collapse postdating other events that removed the bulk topography of this rim section. This proposition agrees with our model of gradual collapse over shallowly buried, thick sequences of volatile-rich materials. The presence of the NW trending grooves (possible faults) highlights the prolonged role of structurally controlled devolatilization during chaotic terrain development. We surmise these hilly surfaces likely include a volatile-free, or less volatile-bearing sedimentary lag produced during the collapse.

## Discussion

### Existence of multiple chaotic terrains on Mercury: evidence of a supraregional volatile-rich crust

Mercury’s most extensive chaotic terrain straddles the antipode of the Caloris basin, but we have discovered smaller occurrences in other regions (Fig. S7A). We present a sample of three chaotic terrain occurrences that are separated by thousands of kilometers (note that this sample does not represent their complete global distribution) (Figs. S7–S11). These chaotic terrains are not at the antipodes of major basins (Table S1), suggesting that they were not formed by the convergence of seismic waves and ejecta. Geomorphologically, all of them share striking similarities. For example, they include evidence of abrupt collapse along crater rims and adjoining plains (yellow arrows in Figs. S7 and S8); the preservation of relatively small craters (~20 km across), highly degraded craters that retain their circularity (white arrows in Figs. S7, S8, S10, S11); and knobs and ridges aligned to regional linear patterns, probably fault-controlled (red arrows in Figs. S7–S10). Furthermore, each region includes apparent zones of incipient, prominent, or extreme landscape modifications, highlighting evidence of the continuum of chaotic terrain development described within the primary study region (Fig. 6). The occurrence of these widespread chaotic terrains supports the existence of deep (sub-kilometer to a couple of kilometers) volatile-rich upper crustal materials with supraregional distributions, which, regionally, underwent gradual and structurally controlled collapse.

### Chaotic terrain development and the formation of local hollows

The global mapping of Mercury’s hollows indicates that they mostly occur within the Low Reflectance Material (LRM) and Low reflectance Blue Plains (LBP) global color units situated in craters and basins, suggesting that these materials might be volatile-rich^34,35^. Our map of the chaotic terrain antipodal to the Caloris basin shows that some of its interior craters enclose smooth floors, which we interpret to be lava emplaced after chaotic terrain development (Fig. 2). The floor of one of these craters (Dario crater), which is located in the easternmost mapped area, contains clusters of hollows that exhibit NW and NE alignments (Fig. S10A,B), the same trends that characterize the chaotic terrains’ dissection patterns (Figs. 4, 5A; S12A,B).

We propose a stratigraphic setting in which the volatile-rich unit that led to hollows formation was not positioned at the surface or near surface, but was instead buried beneath lavas (Fig. 8; S12C). The hollows’ distribution pattern ties their origin to our proposed mechanism of chaotic terrain development due to structurally controlled upper crustal volatile losses (Figs. 8; S12C). We note, however, that chaotic terrain crater interior plains typically lack evidence of collapse, probably because the lava deposits generally prevented the escape of subsurface volatiles. In this geologic context, the origin of the hollows might represent rare fractured floor zones, where emissions of buried volatiles enlarged overlying lava fissures, leading to subsequent localized collapse.

These observations highlight the possibility that the formation of some hollows and the chaotic terrains involved volatile removal from the same buried materials. In this case, these hollows might represent evidence of a geologically recent collapse of Mercury’s volatile-rich upper crust. Here, we do not propose that the above scenario necessarily applies to the origin of hollows in other regions of Mercury. However, we note a relevant aspect of our hypothesis- the upper stratigraphy of the LRM and LBP could consist of geologically young sublimation lags sealing a buried volatile-rich crust, in which hollows formed due to local breaches in the lag deposits. This scenario is attractive because it could reconcile the ancient origin of the volatile-bearing rocks and the fact that hollows appear to be of geologically young development.

### Geologic triggers of collapse and longevity of chaotic terrain formation

We hypothesize that the chaotic terrains could have formed above magmatic hotspots, also accounting for their latitudinal spread, ranging from near-equatorial to subpolar regions (Fig. S7A, Table S1). Widespread plains, probably volcanic, embay the chaotic terrains (Figs. 1A, 2A). Thus, the lava emplacement by the volcanic eruptions must have post-dated the onset of the collapse. This age relationship is consistent with crater counts on the lava plains, which indicate that they formed ~1.9 +/− 0.1 Ga (Fig. 2A,D), virtually the same period estimated for development of the chaotic terrain within the error bars. These temporal relationships suggest that chaotic terrain development mostly occurred during a hotspot thermal anomaly. The proposed volcanic underplating of the volatile rich crust could explain its devolatilization, collapse, and the post-collapse embaying plains could be the last phase of this same volcanic event. It is uncertain how long this phase of volcanic activity could have lasted, but based on Earth cases, it was probably several to a few tens of million years.

The significantly larger size of Mercury’s main chaotic terrain might be a consequence of pervasive fracturing that was inherited much earlier in the planet’s history due to the Caloris impact event^23–27^, and which could have facilitated the upward release of buried volatiles. Furthermore, the distribution of hotspots could be related to large-scale impacts. For example, it has been proposed that the South Pole-Aitken impact mobilized melt around the whole Moon (particularly beneath the antipodal lunar nearside), leading to an epoch of volcanic activity^27^.

The presence of hollows suggests that top-down heat disturbances over lava-covered volatile-rich materials, which extended above the faulted materials, could have resulted in a geologically recent history of collapse in the chaotic terrain’s easternmost part (Fig. S12A,B). This area of the chaotic terrain is also striking in that some of Mercury’s most recent crater rays appear muted or truncated where their traces intersect it (Fig. S13). These observations raise the possibility that increases in solar luminosity over time may have driven episodes of collapse in regions where volatile-rich materials were exposed at the surface or contained within the near-surface.

### Composition and origin of the volatile-rich crust

Multiband visible-near infrared reflectance spectra from the imaging data show absorptions indicative of MgS within hollows^36^. MgS is, however, refractory at temperatures prevailing on Mercury’s surface and upper few kilometers; consequently, it most likely forms a chemical residue from the devolatilization process. Based on investigations of hollows, it appears that the LRM and the LBP might be parts of the volatile-rich crust. While MESSENGER’s spectroscopic observations have not revealed diagnostic mineral identifications^3,37^, chemical data analyses show that Mercury’s crust formed under highly reducing, low oxygen fugacity conditions^38,39^. Under such circumstances, detected lithophile elements Cr, Mn, and Ti^1^ would have become chalcophilic and partitioned into sulfide phases^36^. Mg- and Ca-bearing sulfides could have also been produced in the absence of these elements or Fe^39,40^, which is commensurate with the low measured Fe and high S abundances^39^. The presence and properties of Ca observed in the exosphere^41^ is indicative of calcium oxides and sulfides in the crust^42^. Therefore, it is plausible that non-refractory sulfur compounds formed part of the gaseous phase that was removed from Mercury’s near-surface materials to create the hollows and the chaotic terrains. The origin of these materials could be endogenic (see Supplementary Constraints on the Composition of Mercury’s Volatile-rich Crust). For example, Mercury and Earth could have formed partly from E-chondrite material^43,44^. The partial melting of enstatite chondrites could have yielded an aubritic crust, in which volatiles would have been mainly sulfides^45^. In this geologic scenario, the volatiles that were removed to form both the hollows and the chaotic terrains could have been compositionally similar.

There is another possibility that also deserves attention and further testing: it is conceivable that Mercury accreted a late veneer of carbonaceous chondrite materials^45^. In this case, the potential volatile-bearing rocks in Mercury’s upper crust could also include salts and salt hydrates (chlorides, sulfates, and carbonates), pnictides, sulfosalts, hydrocarbons, and phyllosilicates (see Supplementary Constraints on the Composition of Mercury’s Volatile-rich Crust). The determination of the composition and origin of the volatile-rich crust implied by our results will require an improved understanding of the planet’s surface composition. A potential obstacle is that billions of years of devolatilization might have emplaced widespread sublimation lags, which perhaps now mask an upper crustal volatile-rich composition.

As it has been proposed for geomorphologically similar chaotic terrains on Mars, the supraregional presence of chaotic terrains on Mercury supports a possible origin resulting from localized to regional disturbances within a global volatile-rich crust (see Supplementary Materials for a summary of comparisons between the chaotic terrains of Mercury and Mars). However, as we show in this section, it is clear that the crust-forming volatiles on both planets must be compositionally distinct.

## Summary and Conclusions

Our key results regarding the origin of Mercury’s chaotic terrains include the collapse (1) was gradual and structurally controlled; (2) removed 100 s to 1000 s of meters of surface relief; (3) was the result of near-surface and deep-seated volatiles; (4) left extensive lag deposits; (5) was, at least within the most extensive occurrence, shortly followed by the emplacement of widespread crater interior lavas, (6) was not geographically restricted to the Caloris antipode, and (7) experienced a significant phase of activity ~1.8 Ga for an as-yet uncertain duration. A key morphologic indicator of collapse is the fact that vast linear patterns dissect the chaotic terrain materials and that these patterns include breached crater rim areas that connect to grooved intercrater regions. We hypothesize that magmatic hotspots could have destabilized these materials, leading to crustal collapse events.

Based on the study of the chaotic terrain antipodal to the Caloris basin, we hypothesize that following the effusion of lavas and the formation of the crater interior plains that embay this chaotic terrain’s knobs, the regional heat flow decreased. This change in the crust’s thermal structure led to the end of the main phase of chaotic terrain development in the region. However, we note that it is also possible that the sun’s progressive heating and increased luminosity led to renewed collapse through the sustained, but slow, volatile losses in a top-down fashion.

Our discovery extends the potential presence of volatile-rich materials on Mercury to significant crustal depths and is consistent with the volatile-rich materials having an upper boundary that reaches the near-surface. The presence of hollows and the truncation of crater rays suggest that there might be some geologically recent collapse within the easternmost part of the chaotic terrain antipodal to the Caloris basin, in which case it would have been most likely driven or constrained by solar heating. There are, however, numerous uncertainties regarding the origin of Mercury’s intercrater plains and its history of extensional tectonism that arise from this investigation (see supplementary materials for discussion on uncertainties and future research directions). Our results indicate that the early crust of Mercury could have contained mixtures of hydrated phases, organics, and ices, which have been driven away, dehydrated, or chemically altered by magmatic and solar heating, except for in permanent shadows of polar craters. Metastable and potentially habitable conditions might have developed episodically or transiently within these crustal materials, thus, extending the habitable zone as far inward toward the Sun as Mercury, and in similar places in other solar systems.

## Supplementary information

Supplementary information.
