# Supplementary material for: The Chaotic Terrains of Mercury Reveal a History of Planetary Volatile Retention and Loss in the Innermost Solar System
Source: Sci Rep. 2020 Mar 16;10:4737. doi: 10.1038/s41598-020-59885-5 (PMC7075900; doi:10.1038/s41598-020-59885-5)
Supplement: Supplementary file 1 — Supplementary information. [file 41598_2020_59885_MOESM1_ESM.docx]

The Chaotic Terrains of Mercury Reveal a History of Planetary Volatile Retention and Loss in the Innermost Solar System

J. Alexis P. Rodriguez^1^*, Gregory J. Leonard^2^, Jeffrey S. Kargel^1^, Deborah Domingue^1^, Daniel C. Berman^1^, Maria Banks^1,3^, Mario Zarroca^4^, Rogelio Linares^4^, Simone Marchi^5^, Victor R. Baker^6^, Kevin D Webster^1^, Mark Sykes^1^

^1^Planetary Science Institute, 1700 E Fort Lowell Road, Suite 106, Tucson, AZ 85719, USA.

^2^Department of Planetary Sciences, University of Arizona, Tucson, AZ 85721, USA.

^3^NASA Goddard Space Flight Center, Greenbelt, MD 20771, USA.

^4^External Geodynamics and Hydrogeology Group, Department of Geology, Autonomous University of Barcelona, 08193 Bellaterra, Barcelona, Spain.

^5^Southwest Research Institute, 1050 Walnut St, Suite 300, Boulder, CO 80302, USA.

^6^Department of Hydrology and Atmospheric Sciences, University of Arizona, Tucson, AZ 85721, USA.

*Corresponding Author: [alexis@psi.edu](mailto:alexis@psi.edu)

**TABLE OF CONTENTS**

1. Synthesis of Mapping Approach 3

2. Methodology for Age Determinations based on Crater Count Statistics 4

3. Supplementary Figures 6

4. Crater Ray Patterns over the Chaotic Terrain: Evidence of Possible Geologically Recent Localized Collapse 29

5. Constraints on the Composition of Mercury’s Volatile-Rich Crust 31

6. The chaotic Terrains of Mercury and Mars: An Emerging Paradigm in

Comparative Planetology 50

7. Uncertainties and Future Research Directions 54

**1. Synthesis of Mapping Approach**

We used the Environmental Systems Research Institute’s (ESRI) ArcGIS software (<http://www.esri.com/software/arcgis>) to produce, store, and analyze the digital morphologic maps presented in this article. The digital mapping project included MESSENGER’s global Map Project base map BDR image mosaic product (166 m per pixel; [Mercury MESSENGER MDIS Global base map BDR](https://astrogeology.usgs.gov/search/map/Mercury/Messenger/Global/Mercury_MESSENGER_MDIS_Basemap_BDR_Mosaic_Global_166m)) and global Low Incidence Angle base map image mosaic product (166 m per pixel; [Mercury MESSENGER MDIS base map LOI Global Mosaic](https://astrogeology.usgs.gov/search/map/Mercury/Messenger/Global/Mercury_MESSENGER_MDIS_Basemap_LOI_Mosaic_Global_166m)). The former was prepared as a global 750-nm mosaic illuminated at an average solar incidence angle near 74°. This product is particularly well-suited for the identification and mapping of geomorphological and structural features across a wide range of spatial scales. We overlaid these image mosaics with the MESSENGER global Digital Elevation Model (DEM) product (665m per pixel, ~1m vertical precision; [Mercury MESSENGER Global DEM v2 Oct. 2016](https://astrogeology.usgs.gov/search/map/Mercury/Topography/MESSENGER/Mercury_Messenger_USGS_DEM_Global_665m)). To help recognize regional topographic differences in a variety of chaotic terrain features, we used the DEM in combination with contour lines and a derived hillshade product. The mapping was done at 1:250,000 to 1:2.5M applying a custom Albers equal-area conic projection. Figure images are generally rendered at 1:7M or better, depending on the features that need to be displayed. All elevations that we provide in this article are relative to Mercury’s zero datum.

**2. Methodology for Age Determinations based on Crater Count Statistics**

We used the crater model production function (MPF) and inner solar system chronology of Marchi et al.^1^ to estimate ages for the chaotic terrains and crater interior plains (Fig. 2). The Marchi et al.^1^ MPF is derived from an input impactor size-frequency distribution (SFD) using crater scaling laws and utilizes our most current knowledge and understanding of the impactor populations^2, 3^. Parameters used include a hard rock scaling strength of Y_0_ = 2 x 10^6^ Pa, a target density of 3.4 g/cm^3^, and an impactor density of 2.6 g/cm^3^ (average NEO density). We assumed a uniform target with no layering and no variation of mechanical properties with depth. The Marchi et al.^1^ MPF uses the most probable impact angle of 45° and wide distributions of possible impact speeds on Mercury (~ 20-60 km/s)^1^.

Crater SFDs for fresh craters on the plains and the chaotic terrain were fit to the production function derived from Near-Earth Objects (NEOs). Crater SFD for collapsed craters on the chaotic terrain were fit to the production function derived from Main Belt Asteroids (MBAs) using craters ≥50 km in diameter. Note that this is the same methodology that was used in Denevi et al.^4^ to establish the age of the Caloris basin forming event.

Our SFDs (Fig 2B-D) and model ages (Fig 2C, D) were estimated using craters ≥5 km in diameter. Background secondary craters indistinguishable from primaries are included in the counts as they are included in the production function. Obvious secondary craters that exist in clusters and linear chains were not included.

**References cited in this section**

1. Marchi, S., Mottola, S., Cremonese, G., Massironi, M. & Martellato, E. A new chronology for the moon and Mercury. *Astrophys. J.* **137,** 4936-4948 (2009).

2. Bottke Jr, W. F. e*t al.* Debiased orbital and absolute magnitude distribution of the near-Earth objects. *Icarus* **156,** 399-433 (2002).

3. Bottke Jr, W. F. *et al.* Linking the collisional history of the main asteroid belt to its dynamical excitation and depletion. *Icarus* **179,** 63-94 (2005).

4. Denevi, B. W. *et al.* Chapter 6 in *The geologic history of Mercury* (eds. Solomon, S. C., Nittler, L. R. & Anderson, B. J.) in press (2018).

**3. Supplementary Figures**

**
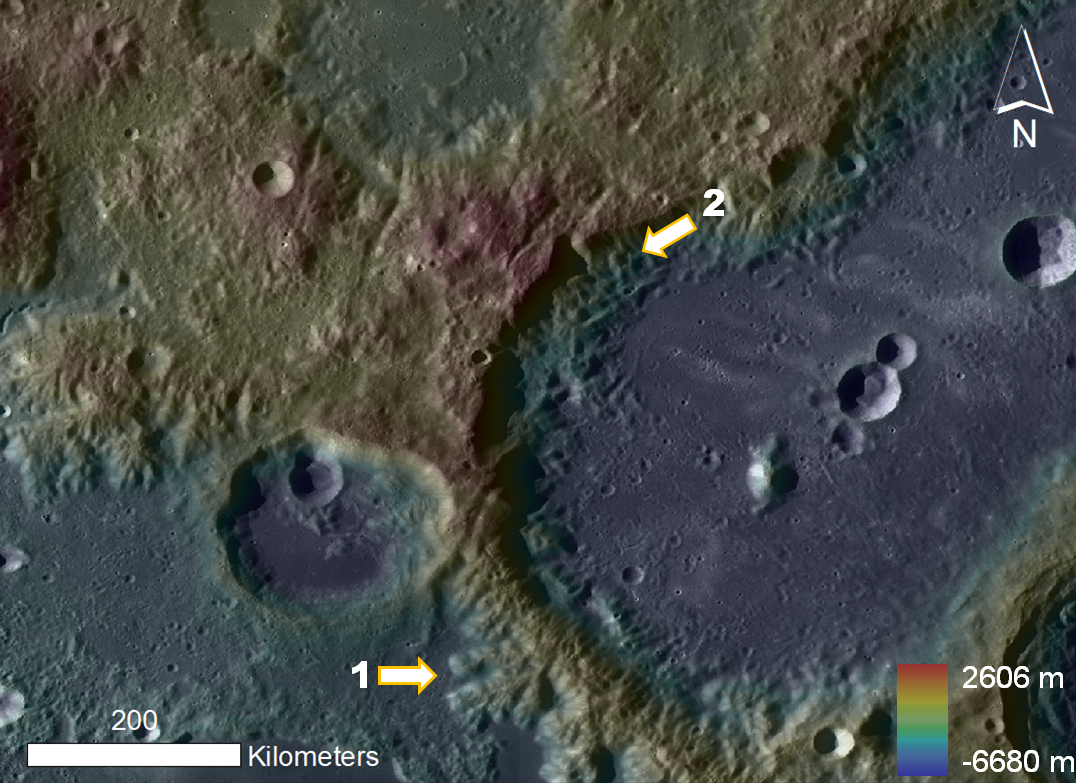
** **Fig. S1** Part of lunar Mare Ingenii (center 30°12'S; 168°13'E) near a proposed seismically disrupted antipode to the Mare Imbrium impact basin. The arrows (1) and (2) respectively identify prominent crater rim-bounding sedimentary lobes and landslides, which exhibit a lineated texture when viewed from orbit. However, there is a stark contrast between the nature of these lineations and those apparent in the crater rims of Mercury’s chaotic terrains, which appear to consist of grooves that cut the rims, in many cases, to their bases (e.g., Figs. 1-5). The image is an LROC (Lunar Reconnaissance Orbiter Camera) Wide Angle Camera (WAC) mosaic (100 m/pixel) over an LRO LOLA DEM (256ppd). Credits to LROC WAC mosaic (http://lroc.sese.asu.edu/about/terms): Robinson, M.S., et al. (2010). Lunar Reconnaissance Orbiter Camera (LROC) Instrument Overview, Space Science Reviews, Vol 150, pp. 81-124. Humm, D.C., et al. (2015). Flight Calibration of the LROC Narrow-Angle Camera, Space Science Reviews Online, pg. 1-43. Mahanti, P. et al. (2015). Inflight Calibration of the Lunar Reconnaissance Orbiter Camera Wide Angle Camera, Space Science Reviews Online, pg. 1-38. Speyerer, E.J. et al. (2012). In-Flight Geometric Calibration of the Lunar Reconnaissance Orbiter Camera, Int. Arch. Photogramm. Remote Sens. Spatial Inf. Sci., XXXIX-B4, pg. 511-516. Robinson, M.S., Lunar Reconnaissance Orbiter Camera Experimental Data Record, LRO-L-LROC-2-EDR-V1.0, NASA Planetary Data System (2010). Credits to LRO LOLA DEM (https://astrogeology.usgs.gov/search/details/Moon/LRO/LOLA/Lunar_LRO_LOLA_Global_LDEM_118m_Mar2014/cub): LOLA Science Team, NASA/GSFC.

**Fig. S2** Part of lunar Mare Ingenii (center 38°17'S, 175°26'E) near a proposed seismically disrupted antipode to the Mare Imbrium impact basin. The interpretation that regional lineated deposits represent thick fallout ejecta debris is consistent with these materials covering crater rims (arrow 1) and forming elevated promontories within inter-crater regions (arrow 2). An important difference within the landscapes of the chaotic terrains of Mercury is the presence of elevated intercrater regions. On Mercury, both the rims and intercrater regions appear to have undergone relief losses (e.g., Figs. 1-5). Also, note that the crater rims are not cut by grooves that are regionally integrated into broad patterns of dissections, as observed on Mercury (e.g., Figs. 1-5). The image is an LROC (Lunar Reconnaissance Orbiter Camera) Wide Angle Camera (WAC) mosaic (100 m/pixel) over an LRO LOLA DEM (256ppd). Credits to LROC WAC mosaic (http://lroc.sese.asu.edu/about/terms): Robinson, M.S., et al. (2010). Lunar Reconnaissance Orbiter Camera (LROC) Instrument Overview, Space Science Reviews, Vol 150, pp. 81-124. Humm, D.C., et al. (2015). Flight Calibration of the LROC Narrow-Angle Camera, Space Science Reviews Online, pp. 1-43. Mahanti, P. et al. (2015). Inflight Calibration of the Lunar Reconnaissance Orbiter Camera Wide Angle Camera, Space Science Reviews Online, pp. 1-38. Speyerer, E.J. et al. (2012). In-Flight Geometric Calibration of the Lunar Reconnaissance Orbiter Camera, Int. Arch. Photogramm. Remote Sens. Spatial Inf. Sci., XXXIX-B4, pp. 511-516. Robinson, M.S., Lunar Reconnaissance Orbiter Camera Experimental Data Record, LRO-L-LROC-2-EDR-V1.0, NASA Planetary Data System (2010). Credits to LRO LOLA DEM (<https://astrogeology.usgs.gov/search/details/Moon/LRO/LOLA/Lunar_LRO_LOLA_Global_L> DEM_118m_Mar2014/cub): LOLA Science Team, NASA/GSFC.

**
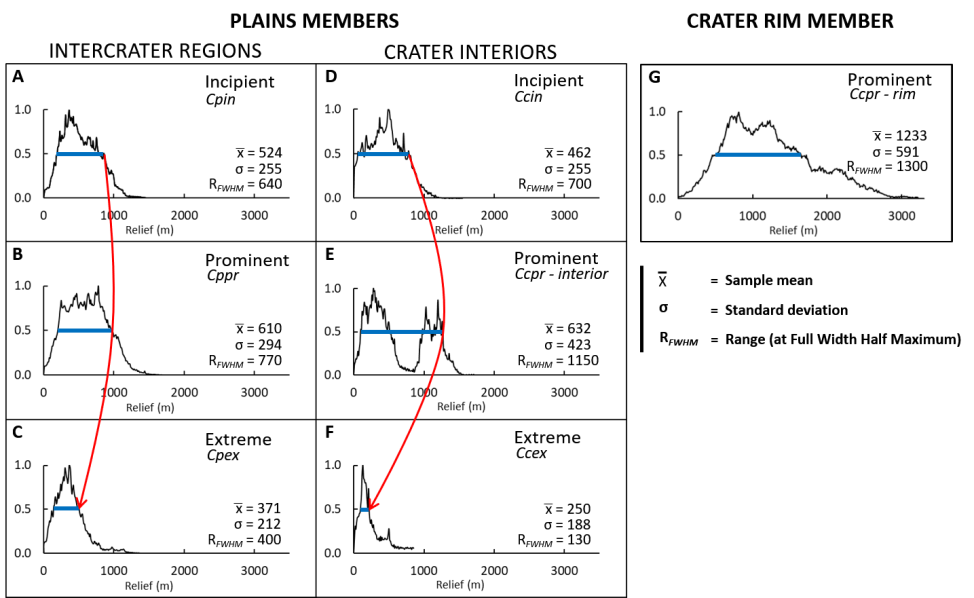
Fig. S3** Relative relief histograms for the Upper Chaotic Terrain Unit (UCTU) members. The elevation bins are 10 m. The x-axes include equivalent relief ranges. The Y-axes, all scaled to unity, show the relief bin frequencies. See map in Fig. 6 for the context and location of each of these members and Table 1 for their descriptions. The intercrater regions plains histograms **(A-C)** show near Gaussian curves; hence, we used full width at half maximum values to determine their comparative ranges (blue lines). The red arrowed line highlights a trend towards an increase in relief range from incipient to prominent terrains, which is consistent with our interpretation that this transition represents a phase of surface collapse into widespread clusters of immense prominent knobs. The trend also reflects a significant decrease in relief ranges from prominent to extreme terrains, again consistent with our interpretation that this transition represents a phase of knob disintegration and overall terrain subdual. A similar trend is apparent in the crater interior region members **(D-F)**. However, we note that the histogram in panel E shows a bimodal distribution, which shows a deficiency in features with relief close to ~1000 m. This bimodal distribution might reflect the prevalence of relatively deeper collapse within these crater interiors floors. **(G)** The crater rim member histogram indicates a greater abundance of high relief features, which is consistent with the rims being extensively breached into ridges and knobs due to the structurally controlled collapse of impact uplifted crustal materials. The wide range in relief values is consistent with a history of gradual collapse (e.g., Fig. S6). Analyses compiled using MESSENGER Mercury global DEM (~665m/px; Credits to: Solomon, S.C. et al. (2001) Planet. Space Sci., 49, 1445-1465.; Hawkins, S.E. III et al. (2007) Space Sci. Rev., 131, 247-338; Becker, K.J. et al. (2016) LPS 47, #2959).


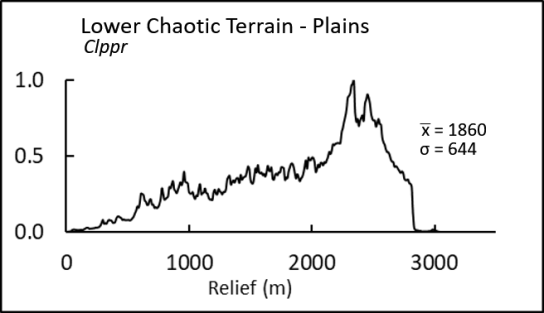


**Fig. S4** Relative relief histogram for the Lower Chaotic Terrain Unit (LCTU). The elevation bins are 10 m. The x-axis shows the relief range. The Y-axis, which is scaled to unity to compare to the relief distributions in the UCTU members directly, shows the relief bin frequencies. See map in Fig. 6 for the unit’s context and location and Table 1 for its descriptions. The histogram shows a non-normal distribution consistent with the collapse of its plains materials producing a higher abundance of high-relief knobs (~2 km) as compared to the UCTU plains materials. This observation is consistent with an overall history of a relatively deeper collapse in the region. Analyses compiled using MESSENGER Mercury global DEM (~665m/px; Credits to: Solomon, S.C. et al. (2001) Planet. Space Sci., 49, 1445-1465.; Hawkins, S.E. III et al. (2007) Space Sci. Rev., 131, 247-338; Becker, K.J. et al. (2016) LPS 47, #2959).

**Fig. S5** As noted in our crater count analysis (Fig. 2), surfaces that retain craters with diameters ≤~10 km are rare within the chaotic terrain. This figure shows a region situated along the chaotic terrain’s boundary (white line; Fig. 1A for location and context). The region adjoining a chaotic terrain’s boundary retains a significant population of these small craters (white arrow). In the adjacent chaotic terrain north of the white line, craters smaller than 10 km are both fewer in number and extremely degraded (e.g., 7 km diameter collapsed crater identified by the blue arrow). We infer that volatiles existed within the rim materials. Solar luminosity could have contributed to their devolatilization, thus diminishing their volume. A system of NW-trending ridges (red arrow) may consist of crater rim remnants and maybe faults that extended to the near-surface. The collapsed craters appear embayed by smooth deposits (yellow arrow), which we interpret as either sedimentary lags emplaced after the volatile loss process or lavas. The panel is a part of a MESSENGER Mercury global DEM (~665 m/px; Credits to: Solomon, S.C. et al. (2001) Planet. Space Sci., 49, 1445-1465.; Hawkins, S.E. III et al. (2007) Space Sci. Rev., 131, 247-338; Becker, K.J. et al. (2016) LPS 47, #2959) draped over MESSENGER Mercury Dual Imaging System (MDIS) global base map (~166 m/px; Credits to: MESSENGER Mercury Dual Imaging System (MDIS) Experiment Data Record (EDR) Software Interface Specification (SIS) document, The Johns Hopkins University, APL, V2T, Jun. 28, 2015; Hawkins, S.E., III, et al., The Mercury Dual Imaging System on the MESSENGER Spacecraft, Space Sci Rev 131: 247–338, DOI 10.1007/s11214-007-9266-3, 2007.; Denevi, B.W. et al., Final calibration and multispectral map products from the Mercury Dual Imaging System Wide-Angle Camera on MESSENGER, Lunar Planet. Sci 47, abstract #1264. NASA/PDS (2016)).

**Fig. S6 (A)** Context and location for high-resolution mosaic RTM N01_002948_0697899 (76 m/px, gray areas). The dashed lines indicate the locations of regional NW and NE structural alignments. **(B)** Close up on the southern part of the RTM, which includes part of a crater’s rim and adjoining plains, which have undergone significant surface elevation losses. The black arrows are within a low albedo region consisting of smooth plains, which occupy the SE part of the image. The white arrows indicate and adjoining higher-albedo zone, containing a rougher surface. These higher albedo materials appear to be exposed in the ejecta of some small craters that occur within the low albedo region, indicating that they form a lower stratigraphic zone (red dots). The high albedo area includes clusters of sub-kilometer-scale hills (e.g., black circle’s interior area). The yellow arrows identify narrow (some sub-kilometer in width) grooves that align with the regional NW structural trends. Our interpretation is that the low albedo surface underwent collapse to produce the high albedo surface and that the collapse involved structurally controlled surface elevation losses. The magnitude of the collapse was variable, and locally there are appear to be low albedo inliers within the collapsed terrains (blue dot). **(A)** View is part of a MESSENGER Mercury global DEM (~665m/px; Credits to: Solomon, S.C. et al. (2001) Planet. Space Sci., 49, 1445-1465.; Hawkins, S.E. III et al. (2007) Space Sci. Rev., 131, 247-338; Becker, K.J. et al. (2016) LPS 47, #2959) draped over MESSENGER Mercury Dual Imaging System (MDIS) global base map (~166m/px; Credits to: MESSENGER Mercury Dual Imaging System (MDIS) Experiment Data Record (EDR) Software Interface Specification (SIS) document, The Johns Hopkins University, APL, V2T, Jun. 28, 2015; Hawkins, S.E., III, et al., The Mercury Dual Imaging System on the MESSENGER Spacecraft, Space Sci Rev 131: 247–338, DOI 10.1007/s11214-007-9266-3, 2007. Denevi, B.W. et al., Final calibration and multispectral map products from the Mercury Dual Imaging System Wide-Angle Camera on MESSENGER, Lunar Planet. Sci 47, abstract #1264. NASA/PDS (2016)). (B) Credit to RTM N01_002948_0697899 goes to the MESSENGER MDIS team and
NASA/JPL/JHU.

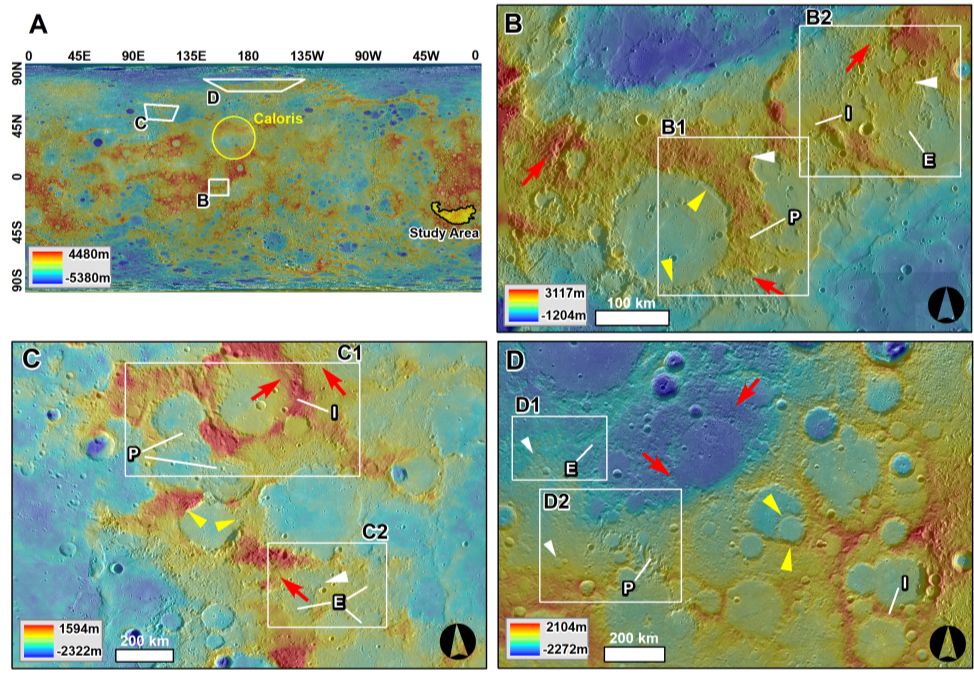
**Fig. S7 (A)** Regions of Mercury showing landscape surface elevation losses that are characteristic of the morphologic zones that we mapped within the chaotic terrain antipodal to the Caloris basin (shown here as yellow shaded ‘Study Area’, and Fig. 6). White arrows indicate relatively small, highly degraded craters, which retain their circularity. The red arrows identify surface elevation losses that exhibit NW and NE structural alignments. The yellow arrows identify rim sections of individual impact craters, which show abrupt and prominent relief changes. The letters I, P, E, respectively, indicate the centers of areas that show geomorphologic indicators of incipient, prominent, and extreme chaotic terrain modifications. All panels are parts of a MESSENGER Mercury global DEM (~665 m/px; Credits to: Solomon, S.C. et al. (2001) Planet. Space Sci., 49, 1445-1465.; Hawkins, S.E. III et al. (2007) Space Sci. Rev., 131, 247-338; Becker, K.J. et al. (2016) LPS 47, #2959) draped over MESSENGER Mercury Dual Imaging System (MDIS) global base map (~166 m/px; Credits to: MESSENGER Mercury Dual Imaging System (MDIS) Experiment Data Record (EDR) Software Interface Specification (SIS) document, The Johns Hopkins University, APL, V2T, Jun. 28, 2015; Hawkins, S.E., III, et al., The Mercury Dual Imaging System on the MESSENGER Spacecraft, Space Sci. Rev. 131, 247–338, DOI 10.1007/s11214-007-9266-3, 2007.; Denevi, B.W. et al., Final calibration and multispectral map products from the Mercury Dual Imaging System Wide-Angle Camera on MESSENGER, Lunar Planet. Sci 47, abstract #1264. NASA/PDS (2016)).

**Table S1** Center latitude and longitude for chaotic terrains shown in Fig. S7A. These chaotic terrains are not antipodal to any significant impact basins.


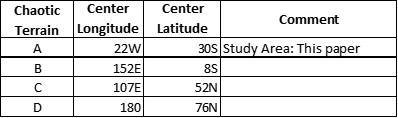


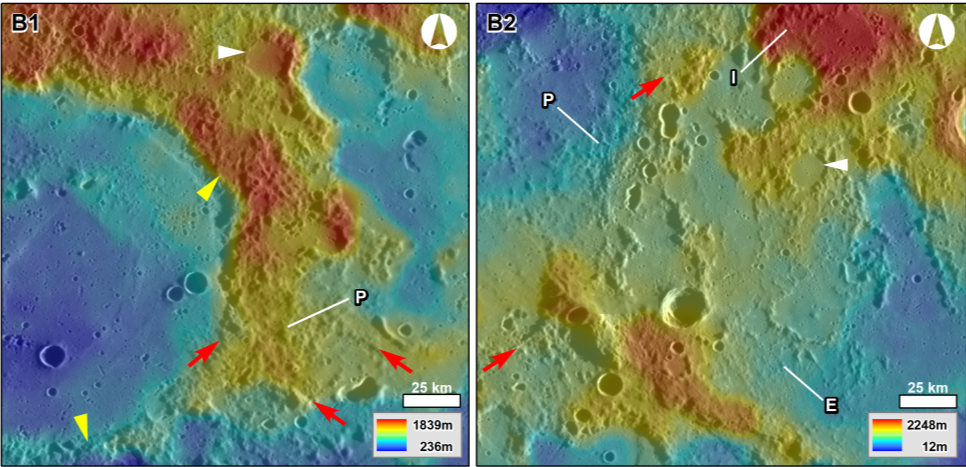
**Fig. S8 (B1 and B2)** Close-up view of subequatorial chaotic terrain occurrences on Mercury. The panels’ contexts and locations are shown in Fig. S7A. White arrows indicate relatively small, highly degraded craters, which have retained their circularity. The red arrows identify surface elevation losses that exhibit NW and NE structural alignments. The yellow arrows identify rim sections of individual impact craters, which show abrupt and prominent relief changes. The letters I, P, E, respectively, indicate the centers of areas that show geomorphologic indicators of incipient, prominent, and extreme chaotic terrain modifications. All panels are parts of a MESSENGER Mercury global DEM (~665 m/px; Credits to: Solomon, S.C. et al. (2001) Planet. Space Sci., 49, 1445-1465.; Hawkins, S.E. III et al. (2007) Space Sci. Rev., 131, 247-338; Becker, K.J. et al. (2016) LPS 47, #2959; draped over MESSENGER Mercury Dual Imaging System (MDIS) global base map (~166m/px; Credits to: MESSENGER Mercury Dual Imaging System (MDIS) Experiment Data Record (EDR) Software Interface Specification (SIS) document, The Johns Hopkins University, APL, V2T, Jun. 28, 2015; Hawkins, S.E., III, et al., The Mercury Dual Imaging System on the MESSENGER Spacecraft, Space Sci Rev 131, 247–338, DOI 10.1007/s11214-007-9266-3, 2007.; Denevi, B.W. et al., Final calibration and multispectral map products from the Mercury Dual Imaging System Wide-Angle Camera on MESSENGER, Lunar Planet. Sci. 47, abstract #1264, NASA/PDS (2016).


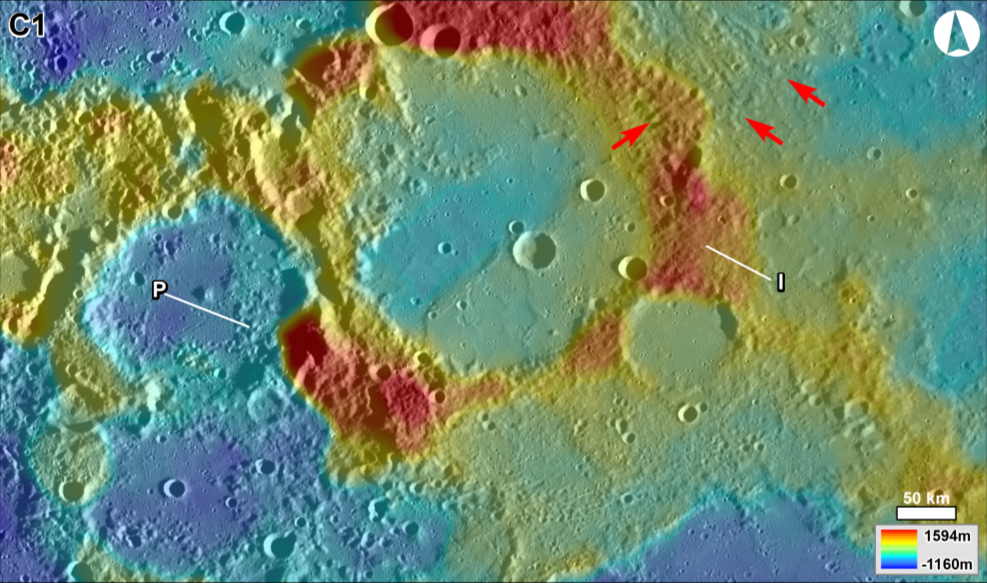


**Fig. S9 (C1)** Close-up view of high latitude chaotic terrain occurrence on Mercury. Context and location are shown in Fig. S7A. The red arrows identify surface elevation losses that exhibit NW and NE structural alignments. The letters I and P, respectively, indicate the centers of areas that show geomorphologic indicators of incipient and prominent chaotic terrain modifications. The base image is part of a MESSENGER Mercury global DEM (~665 m/px; Credits to: Solomon, S.C. et al. (2001) Planet. Space Sci., 49, 1445-1465.; Hawkins, S.E. III, et al. (2007) Space Sci. Rev., 131, 247-338; Becker, K.J. et al. (2016) LPS 47, #2959) draped over MESSENGER Mercury Dual Imaging System (MDIS) global base map (~166 m/px; Credits to: MESSENGER Mercury Dual Imaging System (MDIS) Experiment Data Record (EDR) Software Interface Specification (SIS) document, The Johns Hopkins University, APL, V2T, Jun. 28, 2015; Hawkins, S.E., III, et al., The Mercury Dual Imaging System on the MESSENGER Spacecraft, Space Sci. Rev. 131: 247–338, DOI 10.1007/s11214-007-9266-3, 2007.; Denevi, B.W. et al., Final calibration and multispectral map products from the Mercury Dual Imaging System Wide-Angle Camera on MESSENGER, Lunar Planet. Sci. 47, abstract #1264. NASA/PDS (2016)).


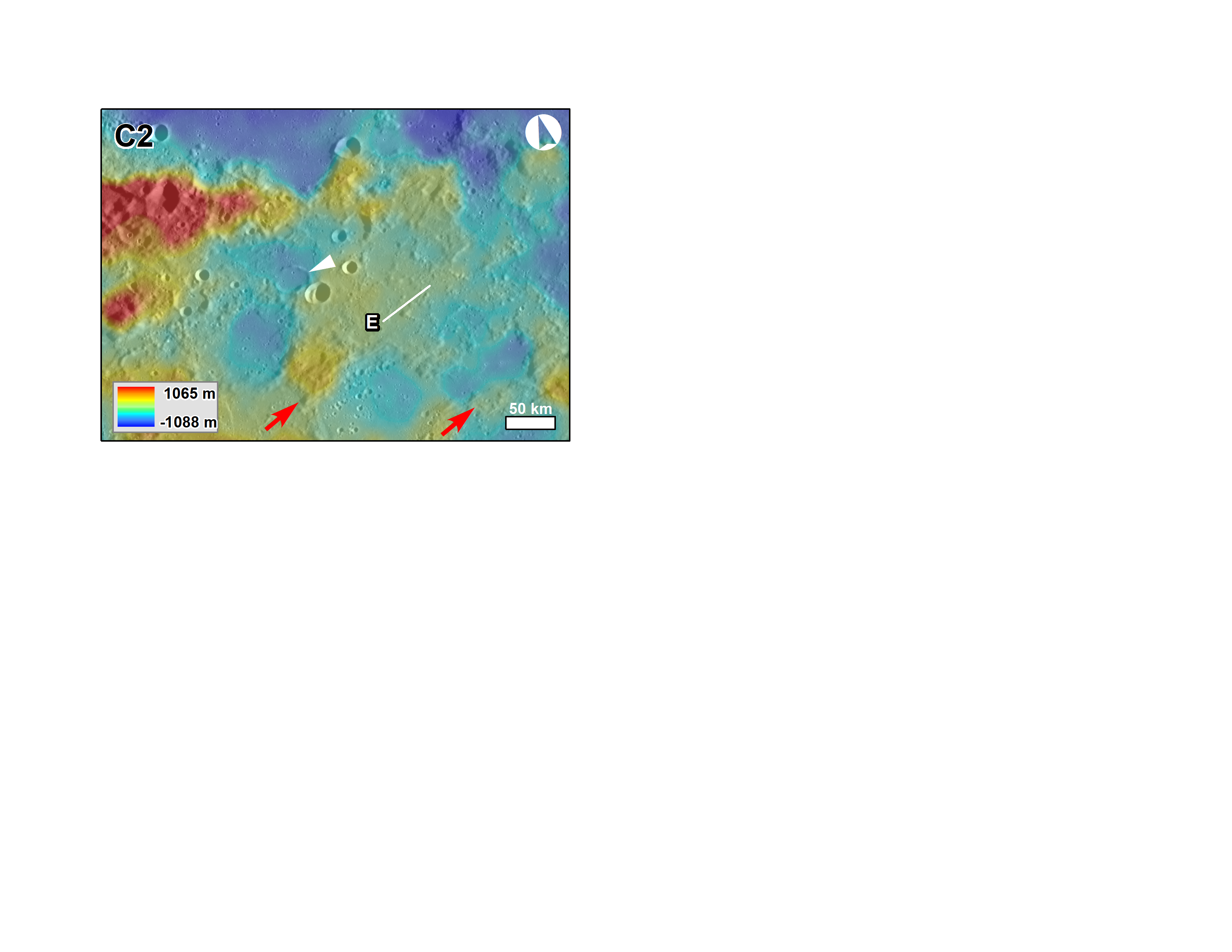


**Fig. S10 (C2)** Close-up view of high latitude chaotic terrain occurrence on Mercury. Context and location are shown in Fig. S7A. The red arrows identify surface elevation losses that exhibit NE structural alignments. The letter E indicates the center of an area that shows geomorphologic indicators of extreme chaotic terrain modifications. White arrow indicates a highly degraded crater, which retains its circularity. The base image is part of a MESSENGER Mercury global DEM (~665 m/px; Credits to: Solomon, S.C. et al. (2001) Planet. Space Sci., 49, 1445-1465.; Hawkins, S.E. III et al. (2007) Space Sci. Rev., 131, 247-338; Becker, K.J. et al. (2016) LPS 47, #2959) draped over MESSENGER Mercury Dual Imaging System (MDIS) global base map (~166 m/px; Credits to: MESSENGER Mercury Dual Imaging System (MDIS) Experiment Data Record (EDR) Software Interface Specification (SIS) document, The Johns Hopkins University, APL, V2T, Jun. 28, 2015; Hawkins, S.E., III, et al., The Mercury Dual Imaging System on the MESSENGER Spacecraft, Space Sci. Rev. 131, 247–338, DOI 10.1007/s11214-007-9266-3, 2007.; Denevi, B.W. et al., Final calibration and multispectral map products from the Mercury Dual Imaging System Wide-Angle Camera on MESSENGER, Lunar Planet. Sci. 47, abstract #1264. NASA/PDS (2016)).


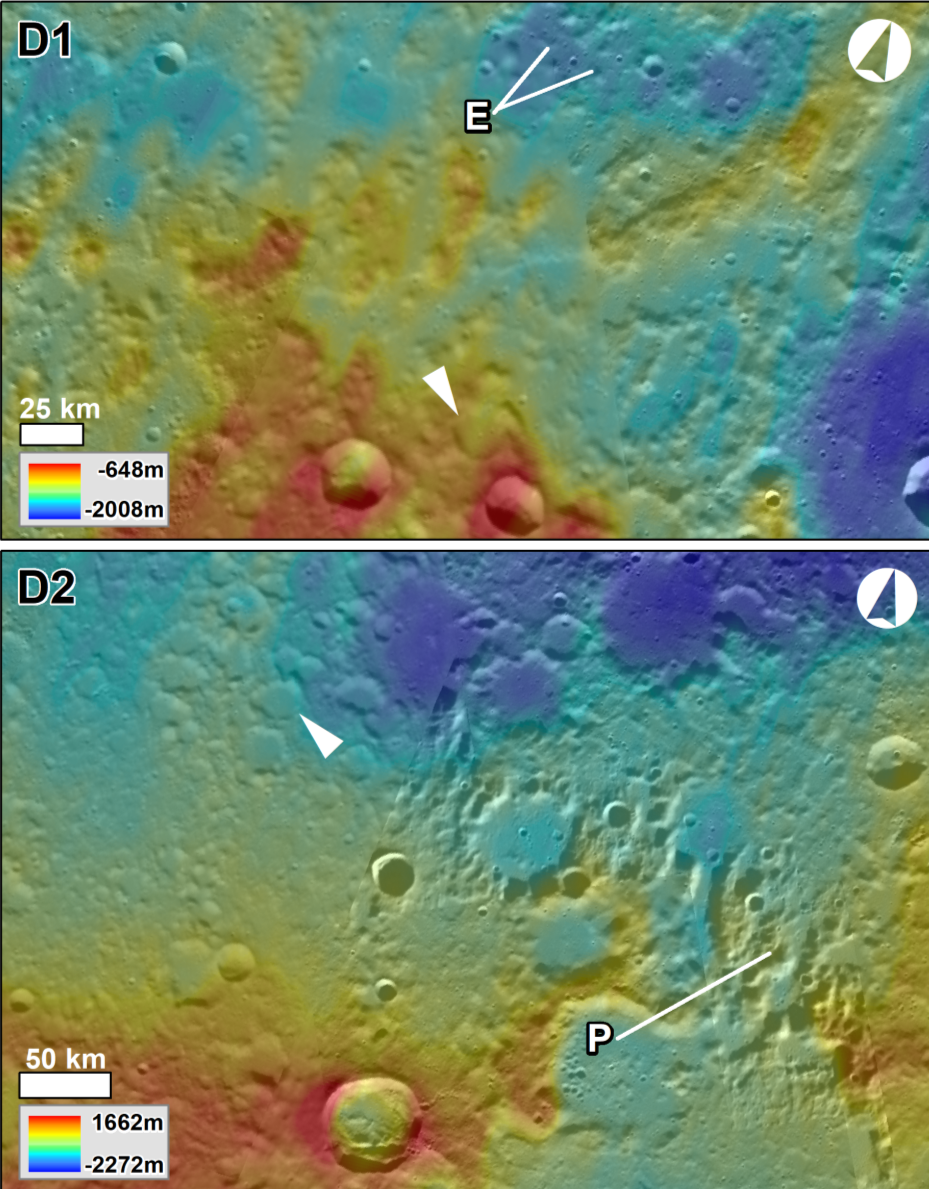
**Fig. S11 (D1 and D2)** Close-up view of circum-polar chaotic terrain occurrence on Mercury. Context and location are shown in Fig. S7A. White arrows indicate relatively small, highly degraded craters, which have retained their circularity. The letters P and E, respectively, indicate the centers of areas that show geomorphologic indicators of prominent and extreme chaotic terrain modifications. The base image is part of a MESSENGER Mercury global DEM (~665 m/px; Credits to: Solomon, S.C. et al. (2001) Planet. Space Sci., 49, 1445-1465.; Hawkins, S.E. III et al. (2007) Space Sci. Rev., 131, 247-338; Becker, K.J. et al. (2016) LPS 47, #2959) draped over MESSENGER Mercury Dual Imaging System (MDIS) global base map (~166m/px; Credits to: MESSENGER Mercury Dual Imaging System (MDIS) Experiment Data Record (EDR) Software Interface Specification (SIS) document, The Johns Hopkins University, APL, V2T, Jun. 28, 2015; Hawkins, S.E., III, et al., The Mercury Dual Imaging System on the MESSENGER Spacecraft, Space Sci. Rev. 131: 247–338, DOI 10.1007/s11214-007-9266-3, 2007.; Denevi, B.W. et al., Final calibration and multispectral map products from the Mercury Dual Imaging System Wide-Angle Camera on MESSENGER, Lunar Planet. Sci. 47, abstract #1264. NASA/PDS (2016)).

**
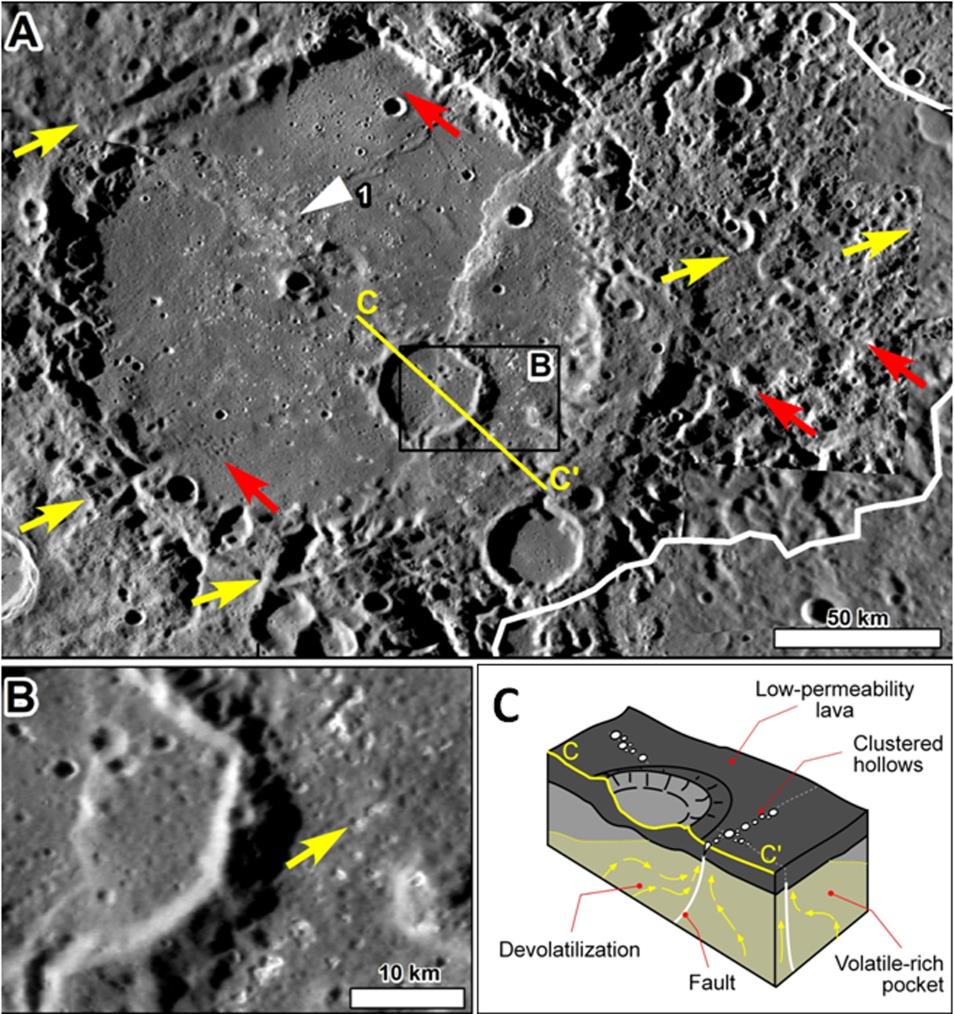
**

**Fig. S12 (A)** View of Dario crater located within the easternmost part of the chaotic terrain antipodal to the Caloris basin. The red and yellow arrows identify rim and interior materials marked by NW and NE trending grooves, respectively. The white line demarks part of the chaotic terrain boundary in the region. The white arrow (1) and panel B’s location box are zones that include hollow clusters. Fig. 1A contains the location and context of this panel. **(B)** Close-up view showing an interior crater, with rims modified by NE-trending collapse. The plains in the view, which embay this crater, exhibit hollows aligned to this orientation. **(C)** Cross-sectional sketch along profile C to C' in panel A. The cross-section illustrates a model in which structurally controlled volatile losses from geologic materials situated beneath the plains resulted in the formation of volatile-rich pockets. Devolatilization of these materials could have produced the observed aligned hollows, in which case their distribution might overlie faults. The base image is part of a MESSENGER Mercury Dual Imaging System (MDIS) global base map (~166 m/px; credits to MESSENGER Mercury Dual Imaging System (MDIS) Experiment Data Record (EDR) Software Interface Specification (SIS) document, The Johns Hopkins University, APL, V2T, Jun. 28, 2015; Hawkins, S.E., III, et al., The Mercury Dual Imaging System on the MESSENGER Spacecraft, Space Sci. Rev. 131, 247–338, DOI 10.1007/s11214-007-9266-3, 2007.; Denevi, B.W. et al., Final calibration and multispectral map products from the Mercury Dual Imaging System Wide-Angle Camera on MESSENGER, Lunar Planet. Sci. 47, abstract #1264. NASA/PDS (2016)).

**4. Crater Ray Patterns over the Chaotic Terrain: Evidence of Possible Geologically Recent Localized Collapse**


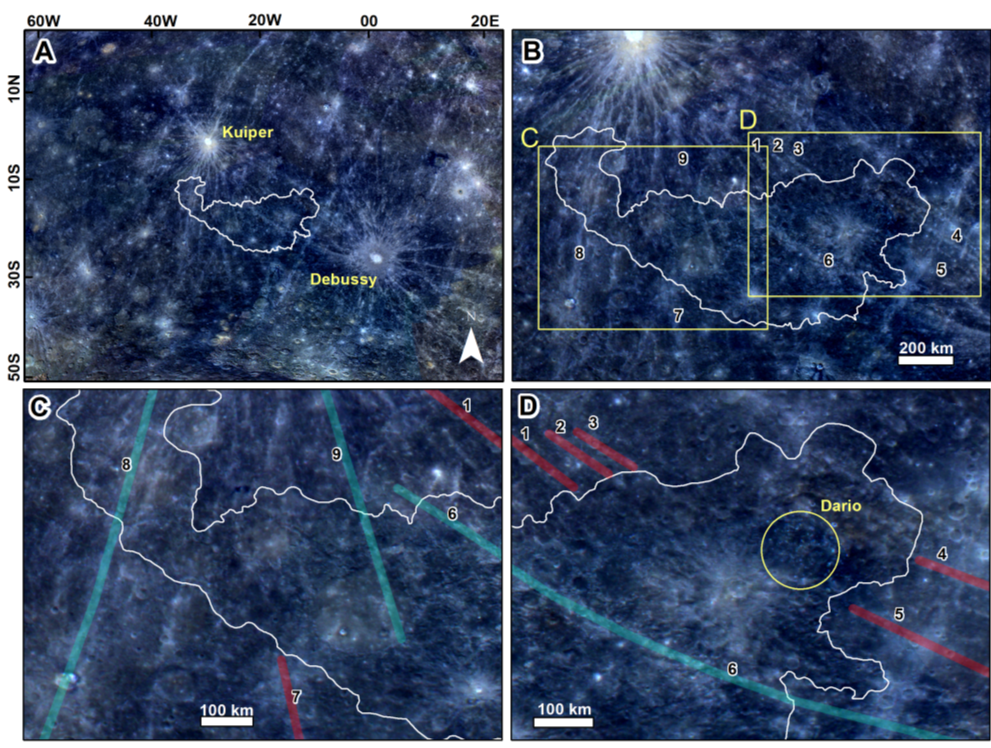
**Fig. S13 (A)** Context view showing the location and extent of the chaotic terrain antipodal to the Caloris basin (outlined in white) relative to the ray systems of the Kuiper and Debussy impact craters. **(B)** Close-up view of panel A that provides the context and locations for panels C and D. The numbers 1-9 identify individual rays within the region’s view. **(C, D)** Close-up view showing crater rays that extend over the chaotic terrain (green lines 6, 8, 9) and other crater rays that appear truncated over the chaotic terrain (red lines 1-5, 7). We provide the location of the hollow hosting crater Dario in panel C (see Fig. S12A). The base image in all panels is a stretched, VNIR false-color composite multispectral image derived from the image product: ‘Mercury MESSENGER MDIS Global Color Mosaic 665m v3’. Credits to: Becker, K. J., et al. (2009). Near Global Mosaic of Mercury: Eos, v. 90, 52, 29 December 2009, Fall Mtg. Suppl., abs. #P21A-1189. <http://abstractsearch.agu.org/meetings/2009/FM/P21A-1189.html>; Hawkins, S. E., III, et al. (2009). In-flight performance of MESSENGER's Mercury Dual Imaging System. Proceedings of the SPIE, v. 7441, pp. 74410Z-74410Z-12. <https://doi.org/10.1117/12.826370>.

The rays of the Kuiper and Debussy impact craters comprise some of the youngest geologic deposits on Mercury^1^. We find that these ray systems exhibit variable continuity over the chaotic terrain antipodal to the Caloris basin (Fig. S13A, B). The rays cover the chaotic terrain’s westernmost part (Fig. S13C). In contrast, the rays are largely truncated over its easternmost part, an area that also includes Dario crater and its interior hollows (Fig. S12). These observations suggest the potential localized to regional removal of surface/near-surface volatiles within the chaotic terrain during geologically recent times, probably driven by solar luminosity.

**References cited in this section**

1. Neish, C. D., Blewett, D. T., Harmon, J. K., Coman, E. I., Cahill, J. T. S., & Ernst, C. M. A comparison of rayed craters on the Moon and Mercury. *J. Geophys. Res. Planets* **118,** 2247–2261 (2013).

**5. Constraints on the composition of Mercury’s Volatile-rich Crust**

In this section, we present constraints on the origin and composition of the proposed volatile-rich crust (VRC) on Mercury. From the morphologic mapping (Fig. 6) and pre-collapse terrain reconstructions (Fig. 7), we estimate a loss of at least ~3.6 x 10^5^ km^3^ of upper crustal materials for Mercury’s largest chaotic terrain — that which is antipodal to the Caloris basin. Assuming a mean density of lost volatile minerals ranging from 1500-3000 kg m^-3^ (encompassing the densities of most volatile sulfides, salts, and phyllosilicates, for instance), the material lost from this chaotic terrain would have had a mass fraction of Mercury’s total mass of 1.6 to 3.3 parts per million.

Our hypothesis involves volatiles that were accumulated widely across Mercury, and then partly lost to form chaotic terrains, and perhaps also the hollows. Below we describe and support two scenarios — each an alternative, but not mutually exclusive, with a goal of creating a set of volatiles that should be considered: (1) *Endogenic model* — The VRC was sourced in Mercury’s mantle and crust. In this case, from the volume of lost material, we infer that the volatile elements had been present in Mercury at abundance levels above roughly 1 ppm; we thus limit our consideration to volatiles where their major elements are contained in Bulk Earth^1^ and enstatite chondrites (two Mercury-analog materials^2, 3^, at abundances >1 ppm), or in aubrites (a Mercury crust analog^4^) at abundances >10 ppm. Less abundant volatiles may have been involved in chaotic terrain formation, but they likely would be of secondary importance with respect to lost volume. (2) *Exogenic model* — a VRC was accreted as a late veneer. In this model, the lost volatiles responsible for chaotic terrain formation must be major constituents of plausible chondrites or comet/Kuiper Belt Objects.

Our geologic observations show that the VRC had an upper boundary that was close to the surface. Hence, solar luminosity, which would have increased over time, likely induced direct top — > down sublimation, or dissociation and vapor loss from the VRC. The observation of chaotic terrains at all latitudes (Fig. S7A) suggests that there is a potential for a wide range of volatiles among the major surface materials. Furthermore, it is likely that there is a latitudinal dependence in the type of volatile composition that has been removed from the VRC, which could have compositionally fractioned the VRC’s surface across latitudinal bands. We will consider these wide-ranging compositions.

**5.1 Relevant Background**

Observations of Mercury’s surface composition support both the endogenic and exogenic models, but the whole-body composition and models of global differentiation pertain more to the endogenic model. McCubbin et al.^5^ first proposed that Mercury is a globally volatile-rich planet, based especially on MESSENGER X-ray spectrometer measurements of K/Th and K/U ratios. Mercury hosts a condensed source of Na, K, Ca, and Mg that are known to supply the exosphere^6-8^. MESSENGER gamma-ray spectroscopy provided detections of Na, K, S, and Cl on the planet’s surface, which exhibit relatively higher concentrations at latitudes >50° N ^9, 10^. Likely candidates for the Cl host — and this may be extended to much of Mercury’s K, Na, and S — include the halides lawrencite (FeCl_2_), sylvite (KCl), and halite (NaCl), as well as Cl-bearing alkali sulfides^10^. Halide and sulfide hosts of Ca, Mg, Na, K, Cl, and S are particularly likely candidates, considering the high abundances of Mg, Ca, Na, K, and S at levels that are not readily explained by the amounts of Si, Al, Fe, Si, and O that would comprise silicates.

Based on the efficiencies of thermal photon, micrometeoroid impact, and solar wind sputtering components of sodium atom losses from the surface and escape and retention efficiencies^8^, we suggest that metastable mineral hosts would likely be mainly cold-spot (mostly polar and circumpolar) condensates. Their formation was likely by reactions of random-walk condensates arriving from lower latitudes and/or polar residues of loss processes. At lower latitudes, Gamborino et al^8^ considered the source reservoirs to be thin chemisorbed layers of native elements, but the fundamental surface supply at those latitudes was thought to be vapor fluxes from the deeper interior. For the more extended, higher altitude exosphere micrometeoroid impact sources were considered likely. Hence, Gamborino et al. ^8^ consider both exogenic and endogenic surface volatile sources of the exosphere.

Mercury’s crust formed under highly reducing, low oxygen fugacity conditions^5, 11^. Under such circumstances, detected lithophile elements Cr, Mn, and Ti^12^ would have become chalcophilic and partitioned into sulfide phases^13, 14^. Mg- and Ca-bearing sulfides could have also been produced in the absence of these elements or Fe^11, 15-17^, which is commensurate with the low measured Fe and high S abundances^11^. The presence and properties of Ca observed in the exosphere^18^ is indicative of calcium oxides and sulfides in the crust^19^. Therefore, with refractory sulfides apparently present, it is plausible that volatile sulfur compounds also occur and formed part of a gas phase^20^ that was evolved and removed from Mercury’s near-surface materials, and then either escaped or partly recondensed near the poles, to create the hollows and the chaotic terrains at subpolar latitudes.

Stockstill-Cahill et al.^21^ made an element balance model of MESSENGER XRF data and separately examined sulfide melts from EH4 enstatite chondrite meteorite Indarch (discussed below) and arrived at average Mercury sulfide compositions ranging between the stoichiometrics of ~Mg_0.65_Ca_0.35_S and ~Mg_0.85_Ca_0.15_S. We note these materials are involatile but could be associated with lost volatiles as the refractory residue of the lost material. MESSENGER’s reflectance spectroscopic observations have not revealed many diagnostic mineral identifications^22, 23^, possibly because of masking by graphite and other darkening agents. Multiband visible-near infrared reflectance spectra from the imaging data show broad absorptions suggestive of MgS or (Mg,Ca)S within hollows^24^. These sulfides are, however, refractory at temperatures prevailing on Mercury's surface and upper few kilometers, and these materials may have accumulated as a devolatilization chemical residue (“lag”).

Vasavada et al^25^ first predicted and modeled the stability of ice in permanently shadowed craters on Mercury. In a stunning confirmation of those predictions, Neumann et al.^26^ and Lawrence et al.^27^, using MESSENGER neutron spectrometer data, found that the northern polar region hosts water ice — likely delivered by comets and asteroids — and that a thin, dark protective insulating layer, perhaps organic materials, covers some polar ice at slightly warmer locations where otherwise it would not remain. Water ice and similarly volatile substances would rapidly disappear on Mercury outside the poles, even at lower solar luminosity, so our search extends beyond ice. Many volatiles could similarly be concentrated near but also well beyond the poles.

**5.2 The Origin and Evolution of the VRC**

**5.2.1. Formation and Nature of Volatile Losses of an Endogenic Volatile-rich crust**

Numerous models and observations point to a potentially long list of suspected sulfides, including MgS and CaS, on Mercury. A leading hypothesis holds that the planet differentiated from an enstatite chondrite (aka E-chondrite) precursor material^27, 28^. Aubrites (i.e., enstatite achondrites thought to be partial melting residues of enstatite chondrites) have some reflectance, petrological, and elemental affinities to Mercury. These meteorites are considered to offer insights into how differentiation of Mercury may have proceeded^29, 30^. E-chondrites represent multiple parent bodies and are major contributors to Earth’s composition^2, 3, 31^ and were probably dominant building blocks for Mercury. They are most noted for containing a large amount of Fe-Ni metal — consistent with Mercury’s huge core mass fraction^32^, as well as refractory silicates, especially MgSiO_3_ (enstatite), and very little iron in the silicates.

E-chondrites and aubrites, apparently like Mercury, are extremely chemically reduced and contain alkali and alkaline earth sulfides: niningerite ((Mg,Fe^2+^,Mn^2+^)S— approximating MgS at low equilibration temperatures), oldhamite ((Ca,Mg)S— approximating CaS at low equilibration temperatures), djerfisherite (K_6_(Fe,Cu,Ni)_25_S_26_Cl) and caswellsilverite (NaCrS_2_), among others.

In melting experiments on EH4 chondrite Indarch, McCoy et al.^33^ show that its sulfides melt near or just below ~1273 K, which they note is ~900 K warmer than the mean annual global surface temperature of Mercury. Hence, MgS and CaS are refractory at temperatures prevailing on Mercury's surface and probably within the upper few kilometers of the crust. We, therefore, propose that Mg-Ca sulfides most likely form a chemical residue from the devolatilization process rather than hosting the volatile.

Clay et al.^34^ deduced three formation mechanisms of djerfisherite and related sulfides on Earth and in the Solar System: (a) primitive djerfisherite as a product of nebula condensation in the unequilibrated E-chondrites; (b) formation by extensive K-metasomatism; and (c) as a product of primary magma unmixing due to silicate-sulfide immiscibility. To these mechanisms must be added (d) impact melting of enstatite chondrites^27, 28^. Those investigators deduced the occurrence of multiple impact melting events on an enstatite chondrite parent body from ~4.5 to ~4.2 Ga; they note that Mercury probably was not the host planet. Those meteorites contain — along with Fe-poor enstatite, Na-plagioclase, and Fe-Ni metal — a range of sulfide and phosphide minerals — including troilite (FeS), niningerite (MgS), daubréelite (FeCr_2_S_4_), oldhamite (CaS), caswellsilverite (NaCrS_2_), djerfischerite (K_3_(Na,Cu)(Fe,Ni)_12_S_14_) and schriebersite ((Fe,Ni)_3_P) — and also graphite. This could be a mineral assemblage native to Mercury. All of these cases could involve magma unmixing (mechanism c); while none of these minerals are volatile under Mercury surface conditions, they are volatile under magmatic conditions and thus could form the residue portion of an exhalative VRC.

Sulfide formation in enstatite chondrite impact melts also was studied by Piani et al.^35^. Their findings indicate that, most likely, as Mercury was heated during and after accretion, a sequence of immiscible sulfidic and metallic melts was generated, which would have sunk to the center, the metals forming a core, and some of the sulfides forming a layer surrounding the core. The silicates and sulfides would have further differentiated as the system heated and cooled. Sulfur also dissolves in the silicate melt, according to the Indarch melting experiments, so continued exsolution of sulfidic liquids and precipitation of sulfide minerals would occur as the crust and mantle cooled. These sulfides then would have remained in the crust and mantle, available to participate in further igneous or impact melting and remixing processes.

Therefore, we propose that the most effective endogenic means of producing the proposed VRC is by magmatic exhalative activity. The observed evidence of volcanic plains emplacement immediately after VRC formation is supportive of longer-lived magmatic hotspots that likely initiated and drove geothermal devolatilization and partial collapse of the VRC.

Moynier et al.^31^ found that enstatite chondrites have the same zinc isotope composition as Earth— another line of evidence, among many others that these meteorites comprise a major ingredient of the terrestrial planets. That team also found that aubrites obtained their zinc by condensation of a zinc-bearing vapor that was lost from the EH6 parent body by thermal metamorphism. Hence, there is not only a melting relationship of enstatite chondrites, but also a devolatilization/sublimation condensation relationship. Their work suggests the likelihood that other elements would have behaved similarly. The same is likely true of Mercury, where volatiles from depth would have recondensed in the upper crust or on the surface, with some volatiles gradually migrating toward higher latitudes as the chaotic terrain collapse proceeded.

**5.4.2. Formation and Nature of Volatile Losses of an Exogenic Volatile-rich Crust**

Mercury may have accreted a late veneer of carbonaceous chondrite materials^4^. In this case, these materials could have formed or contributed to the formation of the VRC. Compositionally, this VRC would have contained salts and salt hydrates (chlorides, sulfates, and carbonates), pnictides, sulfosalts, hydrocarbons, phyllosilicates. Highly hydrated carbonaceous chondrites, for example, undergo substantial water vapor (and other volatile) evolution with large concurrent volume losses, but these changes are generally minor until temperatures of 673-873 K are reached^36^ — above the Mercury surface and upper crust temperatures. Consequently, these materials would not only be partially volatile under Mercury’s surface and upper crust conditions, but they would also be chemically reactive with respect to native Hermean rocks. The results of such reactions can be the production of water vapor and other volatiles, refractory graphite, and large volume reductions of the solid residues. For example, highly labile elements and molecules, such as H_2_O, Se, As, and Sb, and small amounts of S are mobilized in some carbonaceous chondrites at temperatures similar to Mercury’s near-surface, e.g., 473 K, but for the most part, the major mobilization of these elements occurs above 600 K^37^.

If high temperatures were attained by magmatism, then the upper crust could devolatilize. Hence, a VRC containing carbonaceous chondrite material would likely remain stable until an episode of global or regional volcanism and underplating of the VRC by magma. If, however, the sun is the primary heat source driving VRC devolatilization, it is plausible that volatiles within the VRC were ices delivered by the impacts of Kuiper Belt Objects, upon which a liquid/vapor-rich aqueous condition would develop in the immediate aftermath of a late accretion event.

As noted above, CaS, though not highly volatile, may exist as surface residue and therefore be indicative of magmatic sourced compounds contributing to the development of the VRC. A natural and industrial means of producing CaS is by carbothermic reduction of calcium sulfate, an idealized stoichiometry being: CaSO_4_ + 2C → CaS + 2CO_2._ The reaction is strongly pressure- and temperature-dependent and proceeds rapidly between ~500-800 K at 100 kPa; and ~400-600 K at 50 Pa^38^. Related carbothermic reductions include Na_2_SO_4_ + 2C → Na_2_S + 2CO_2 ._ Worden et al.^39^ showed that similar reactions (thermochemical sulfate reduction, TSR) occur on Earth in geological environments— natural gas or petroleum reduction of evaporitic sulfates— at a lower temperature limit of 413 K; another study found a similar geological environment where the reactions occurred starting at a temperature near 403 K^40^ . Graphite, coal, natural gas, petroleum, or carbon monoxide can be the reductant driving TSR of Ca-, Mg-, Na-, and K-sulfates. Some reactions require a solid catalyst or helper gas; for instance, when an iron mineral catalyst is present, CaS can be produced by CaSO_4_ (s) + 4CO(g) —> CaS(s) + 4CO_2_ (g). The reactions proceed in pressurized aqueous environments also, but then they commonly yield hydrogen sulfide gas. The products of these reactions include sulfides of the alkalis and alkaline earth metals^41^; volatiles, including H_2_S, CO_2_; a wide range of volatile organic molecules including highly volatile mercaptan and thiophene and less volatile long-chain alkanes; and refractory organics^40, 42,43^ .

TSR, like the idealized carbothermic reactions above, operates at temperatures similar to the global mean annual surface temperature of Mercury. However, it needs a reductant and an oxidized feedstock. Graphite is likely to be abundant deep within the Hermean crust and locally on the surface^44^. Furthermore, the same unequilibrated carbonaceous asteroids and comets that bring in polar volatiles likely also add reduced organic materials globally and could bring in sulfates and other oxidized materials. Sulfates also could be produced locally in impact-heated oxygen-rich environments due to impact dissociation of water (and hydrogen loss) from hydrous or icy asteroids or comets. The highly chemically disequilibrated nature of some carbonaceous chondrites and comets or KBOs would be aggravated by ingestion into the ultra-reduced, warm Hermean crust.

Of the djerfisherite formative mechanisms mentioned in the section above, on Earth, djerfisherite is known also from the natrocarbonatite volcano Oldoinyo L’engai (i.e., molten salt volcano), where multi-liquid immiscibility (mechanism (c) in the list of djerfisherite forming mechanisms) has taken place in Earth's mantle or during magma ascent^45^. We suggest that TSR or carbothermic reduction is a likely origin of Oldoinyo L’engai’s sulfides, with liquid-liquid immiscibility then occurring. Mercury apparently has a graphite-rich crust, and the volatiles known on the planet would likely include some— sulfate salts especially — that react with graphite under Mercury near-surface temperatures. We propose that this is a possible origin of its MgS and CaS if the exogenic model of the VRC is correct. Similar chemical reactivity would generate a host of other sulfides, some highly volatile.

**5.3. Synthesis**

The discussion above indicates wide-ranging possibilities for the chief VRC volatiles and residual derivatives. We have examined vapor pressures, solid densities, and melting temperatures of some pure volatile phases (and related non-volatile materials). Figure S14 shows these properties for a wide range of substances. For endogenic models, especially those involving magmatic flotation, favored surface volatiles would have low melting points and low densities. For those involving endogenic magmatic-exhalative deposition, favored volatiles have high vapor pressures and maybe low melting temperatures and low densities. For exogenic models, condensation — hence high vapor pressure — is most crucial. For instance, note that NaCl — a plausible host of polar-concentrated Na and Cl on Mercury and also a likely magmatic exhalative condensate — has a volatility similar to ice on Callisto, where hot-point ice sublimation and cold-trap condensation occurs^46^. Chlorides and sulfates, if present, would form hydrates (not shown in the figure) if a chondritic veneer was involved in the VRC, and those have very large volume reductions at relatively low temperatures. In anhydrous form, these salts generally have a range of low densities, low melting points (when present in salt mixtures), and high vapor pressures to be consistent with solar-heated mobilization and polar deposition on Mercury. Alkali sulfides are volatile and have low densities and could participate in VRC and chaotic terrain development. The alkaline earth sulfides CaS and MgS — much considered in the Mercury literature — have low densities in magmatic systems and they could float and be a component of the VRC, but they are not very volatile; hence, Ca-Mg sulfides would tend to form lag residues. Many organics, upon surface exposure to solar radiation or the low-grade metamorphic temperatures of Mercury’s upper crust, would convert to high-mass carbonaceous residues — graphite in the extreme case, consistent with suggestions that it is present on the surface of Mercury. Graphite thus has both exogenic and endogenic means of formation and would be a lag material, but some volatiles would sublimate and redeposit at high latitudes. Most transition metal sulfides, as shown, are very dense and involatile, and also are miscible in Fe-Ni metal; they likely are mainly partitioned into Mercury’s core and do not likely have a role in the VRC and chaotic terrain development. As mentioned in the section above, phyllosilicates (not shown in the figure) could participate in the formation and devolatilization of the VRC if devolatilization occurred by high temperatures caused by magmatic underplating; some of the evolved water vapor could redeposit at the poles.

Additional information, perhaps from a future mission to Mercury, is required to further narrow geochemical constraints on the composition and, therefore, origins for Mercury’s VRC and chaotic terrain development.

*
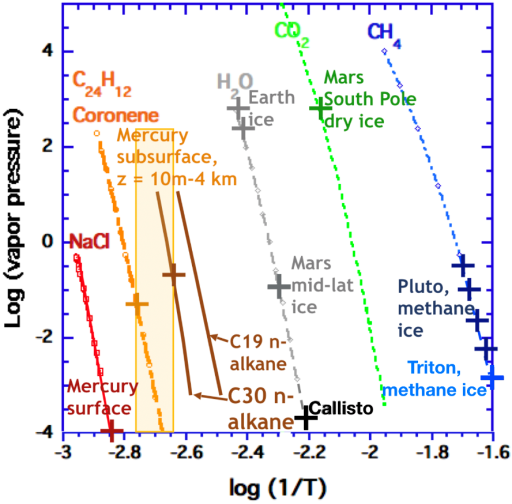
*

*
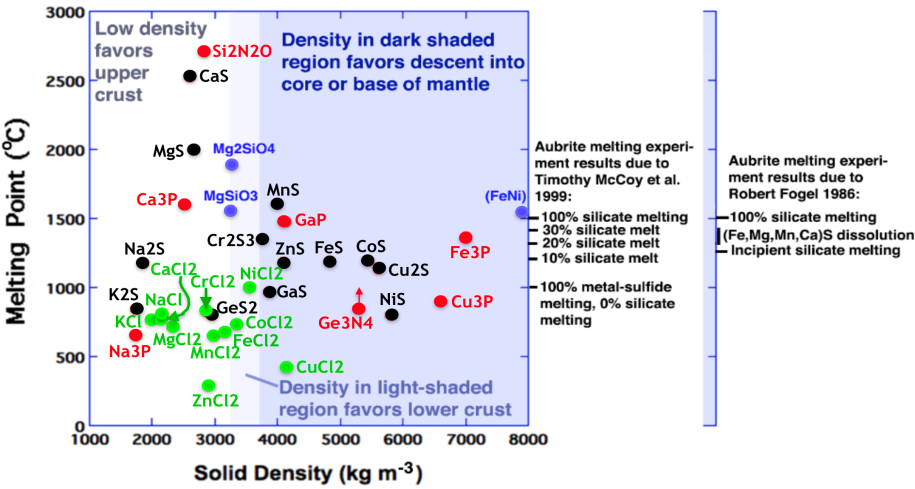
*

**Fig. S14** **(Above)**: Vapor pressure vs. inverse temperature for a representative salt (NaCl), organics, compared with H_2_O, CO_2_, and CH_4_ ices. Planetary surface temperatures are indicated by + signs. The vertical orange region is Mercury’s crustal temperatures (calculated) ranging from 10 m to 4 km deep. **(Below)**: Melting points and solid densities of sulfides (black), chlorides (green), pnictides (red), and major phases in aubrites (blue). Partial melting conditions found for aubrite meteorites are at the far right^33, 47^.

**References cited in this section**

1. Kargel, J. S, & Lewis, J. S. The composition and early evolution of Earth. *Icarus* **105**, 1-25 (1993).

2. Lodders, K. Alkali elements in the Earth's core: Evidence from enstatite meteorites. *Meteoritics and Planetary Science* **30**, 93-101 (1995).

3. Kargel, J. S. A possible enstatite meteorite-Earth connection and potassium in Earth’s core. *Meteoritics and Planetary Science* **30**, 93-101, 5-7 (1995).

4. Braukmuller, N., Wombacher, F., Funk, C. & Munker, C. Earth’s volatile element depletion pattern inherited from a carbonaceous chondrite-like source. *Nature Geoscience* **12**, 564-568 (2019).

5. McCubbin, F. M., Riner, M. A., Vander Kaaden, K. E. & Burkemper, L. K. Is Mercury a volatile-rich planet? *Geophys. Res. Lett*. **39**, L09202 (2012).

6. Ip, W. H. The sodium exosphere and magnetosphere of Mercury. *Geophys. Res. Lett.***13**, 423–426. https://doi.org/10.1029/GL013i005p00423 (1986).

7. Sprague, A. L. Mercury’s atmospheric bright spots and potassium variations: A possible cause. *J. Geophys. Res. Planets* **97(E11)**, 18257–18264. https://doi.org/10.1029/92JE01690 (1992).

8. Gamborino, D., Vorburger, A. & Wurz, P. Mercury’s subsolar sodium exosphere: an ab initio calculation to interpret MASCS/UVVS observations from MESSENGER. *Ann. Geophys.* **37**, 455–470. <https://doi.org/10.5194/angeo-37-455-2019> (2019).

9. Peplowski, P. N. *et al.* Variations in the abundances of potassium and thorium on the surface of Mercury: Results from the MESSENGER Gamma-Ray Spectrometer. *Jour. Geophys. Res.* **117**, E00L04, https://doi.org/10.1029/2012JE004141 (2012).

10. Evans, L .G. *et al.* Chlorine on the surface of Mercury: MESSENGER gamma-ray measurements and implications for the planet’s formation and evolution. *Icarus* **257**, 417-427 (2015).

11. Vander Kaaden, K. E. *et al.* Geochemistry, mineralogy, and petrology of boninitic and komatiitic rocks on the mercurian surface: Insights into the mercurian mantle. *Icarus* **285,** 155-168 (2017).

12. Nittler, L. R. *et al.* The Major-Element Composition of Mercury’s Surface from MESSENGER X-ray Spectrometry. *Science* **333,** 1847-1850 (2011).

13. Vilas, F. *et al.* Mineralogical indicators of Mercury’s hollows composition in MESSENGER color observations. *Geophys. Res. Lett*. **43,** 1450-1456 (2016).

14. Cartier, C. & Wood, B. J. The role of reducing conditions in building Mercury. *Elements* **15**, 39–45.<https://doi.org/10.2138/gselements.15.1.39>. (2019).

15. Vander Kaaden, K. E. & McCubbin, F. M. The origin of boninites on Mercury: An experimental study of the northern volcanic plains lavas. *Geochim. Cosmochim. Acta* **173,** 246-263 (2016).

16. Margot, J. -L., Hauck II, S. A., Mazarico, E., Padovan, S. & Peale, S. J. Mercury’s internal structure, in: *Mercury - The View after MESSENGER*, S. C. Solomon, B. J. Anderson, L. R. Nittler (editors), arXiv:1806.02024 [astro-ph.EP]. (2018).

17. Zolotov, M. *et al.* The redox state, FeO content, and origin of sulfur-rich magmas on Mercury. *J. Geophys. Res. Planets* **118**, 138–146. https://doi.org/10.1029/2012JE004274. (2013).

18. Killen, R. S., Bida, T. A. & Morgan, T. H. The calcium exosphere of Mercury. *Icarus* **173,** 300-311 (2005).

19. Killen, R. S. Pathways for energization of Ca in Mercury’s exosphere. *Icarus* **268,** 32-36 (2016).

20. Zolotov, M. Tu. On the chemistry of mantle and magmatic volatiles on Mercury. *Icarus* **212**, 24–41 (2011).

21. Stockstill-Cahill, K. R., McCoy, T. J., Nittler, L. R. & Weider, S. Magnesium-rich compositions on Mercury: Implications for magmatism from petrologic modeling. *43rd Lunar and Planetary Science Conference*, abstract #2107 (2012).

22. Murchie, S. L. *et al.* Orbital multispectral mapping of Mercury with the MESSENGER Mercury Dual Imaging System: Evidence for the origins of plains units and low-reflectance material. *Icarus* **254,** 287-305 (2015).

23. Izenberg, N. R. *et al.* The low-iron, reduced surface of Mercury as seen in spectral reflectance by MESSENGER. *Icarus* **228,** 364-374 (2014).

24. Vilas, F. *et al.* Mineralogical indicators of Mercury’s hollows composition in MESSENGER color observations. *Geophys. Res. Lett*. **43,** 1450-1456 (2016).

25. Vasavada, A. R., Paige, D. A. &Wood, S. E. Near-surface temperatures on Mercury and the Moon and the stability of polar ice deposits. *Icarus* **141**, 179-193 (1999).

26. Neumann, G. A. *et al.* Bright and dark polar deposits on Mercury: Evidence for surface volatiles. *Science* **339**, 296-300  [https://doi.org/10.1126/science.1229764](%20https://doi.org/10.1126/science.1229764) (2013).

27. Lawrence, D. J. *et al.* Evidence for water ice near Mercury’s north pole from MESSENGER neutron spectrometer measurements. *Science* **339**, 292-296 (2013).

.27. Wilbur, Z. W. *et al.* Aubrite and enstatite chondrite impact melt meteorites: Analogs to Mercury? *49th Lunar and Planetary Science Conference* **2018**, abstract 1355 (2018).

28. Udry, A., Wilbur, Z. E., Rahib, R. R. & McCubbin, F. M. Reclassification of four aubrites as enstatite chondrite impact melts: Potential geochemical analogs for Mercury. *Meteoritics and Planetary Science* **54**, 785-810<https://doi.org/10.1111/maps.13252>. (2019).

29. Keil, K. Enstatite achondrite meteorites (aubrites) and the histories of their asteroidal parent bodies. [*Chemie der Erde - Geochemistry*](https://www.researchgate.net/journal/0009-2819_Chemie_der_Erde-Geochemistry) **70**, 295-317 https//doi.org/[10.1016/j.chemer.2010.02.002](http://dx.doi.org/10.1016/j.chemer.2010.02.002) (2010).

30. Burbine, T. H. *et al.* Spectra of extremely reduced assemblages: Implications for Mercury. *Meteoritics & Planetary Science* **37**, 1233–1244 (2002).

31. Moynier, F. *et al.* Nature of volatile depletion and genetic relationships in enstatite chondrites and aubrites inferred from Zn isotopes. *Geochim. Cosmochim. Acta* **75**, 297-307 (2011).

32. Margot, J.-L., Hauck II, S. A., Mazarico, E., Padovan, S. & Peale, S. J. Mercury’s internal structure, in: *Mercury - The View after MESSENGER*, S. C. Solomon, B. J. Anderson, L. R. Nittler (editors), arXiv:1806.02024 [astro-ph.EP] (2018).

33. McCoy, T., Dickinson, T. & Lofgren, G. Partial melting of the Indarch (EH4) meteorite: A textural, chemical and phase relations view of melting and melt migration. *Meteoritics & Planetary Science* **34**, https://doi.org/10.1111/j.1 (1999).

34. Clay, P. L., O’Driscoll, B., Upton, B. G. J. & Busemann, H. Characteristics of djerfisherite from fluid-rich, metasomatized alkaline intrusive environments and anhydrous enstatite chondrites and achondrites. *American Mineralogist* **99**, 1683-1693. (2014).

35. Piani, L., Marrocchi, Y., Libourel, G. & Tissandier, L. Magmatic sulfides in the porphyritic chondrules of EH enstatite chondrites.*Geochimica et Cosmochimica Acta* **195**, 84-99 (2016).

36. King, A. J. *et al.* The alteration history of the Jbilet Winselwan CM carbonaceous chondrite analog for C-type asteroid sample return. *Meteoritics & Planetary Science* **54**, 521-543 (2019).

37. Nakamura, T. Review: Post-hydration thermal metamorphism of carbonaceous Wordenchondrites. *Jour. Mineralogical and Petrological Sciences* **100**, 260-272 (2005).

38. Yan, *et al.* Decomposition of calcium sulphate by carbothermic reduction at reduced pressures. *Jour. Vacuum Science and Technology* **34**, 517-521. https://doi.org/ [10.3969/j.issn.1672-7126.2014.05.15](http://dx.doi.org/10.3969/j.issn.1672-7126.2014.05.15) (2014).

39. Worden, R. H., Smalley, P. C., and Oxtoby, N. H. Gas souring by thermochemical sulfate reduction at 140°C. *AAPG Bulletin* **79**, 854-863 (1995).

40. Cai, C., Worden, R. H., Bottrell, S. H., Wang, L. & Yanga, C. Thermochemical sulphate reduction and the generation of hydrogen sulphide and thiols (mercaptans) in Triassic carbonate reservoirs from the Sichuan Basin, China. *Chemical Geology* **202**, 39–57 (2003).

41. Ding, K., Wang, S., Li, S. & Yue, C. Thermochemical reduction of magnesium sulfate by natural gas: Insights from an experimental study. *Geochemical Journal* **45**, 97-108 (2011).

42. Kelemen, S. R. *et al.* Distinguishing solid bitumens formed by thermochemical sulfate reduction and thermal chemical alteration. *Organic Geochemistry* **39**, 1137-1143 (2008).

43. Hoşgörmez, H., Yalçın, M. N., Soylu, C. & Bahtiyar, I. Origin of the hydrocarbon gases carbon dioxide and hydrogen sulfide in Dodan Field (SE-Turkey). *Marine and Petroleum Geology* **57**, 433-444 (2014).

44. Peplowski, P. N. *et al.* Remote sensing evidence for an ancient carbon-bearing crust on Mercury. *Nature Geoscience* htps://doi.org/10.1038/NGEO2669 (2016).

45. Sekisova, V. S., Sharygin, V. V., Zaitsev, A. N. & Strekopytov, S. Liquid immiscibility during crystallization of forsterite-phlogopite ijolites at Oldoinyo Lengai Volcano, Tanzania: study of melt inclusions. [Russian Geology and Geophysics](https://www.sciencedirect.com/science/journal/10687971) **56**, 1717-1737 <https://doi.org/10.1016/j.rgg.2015.11.005> (2015).

46. White, O. L., Umurhan, O. M., Moore, J. M. &Howard, A. D. Modeling of ice pinnacle formation on Callisto. *Jour. Geophys. Res. Planets* **121**, 21-45 (2016).

47. Fogel, R. A., Hess, P. C. & Rutherford, M. J. The enstatite chondrite-achondrite link. *Lunar and Planetary Science* XIX, 342-343; and work, unpublished, in 1986. (1988).

**6. The chaotic Terrains of Mercury and Mars: An Emerging Paradigm in Comparative Planetology**

The term “chaotic terrain” was first used to describe complex, topographically collapsed, and disintegrated areas of Mars^1^. These terrains are thought to be residual broken up crust that formed over regions of enormous groundwater outflows^1-9^. We find that Mercury’s chaotic terrains exhibit striking geomorphologic similarities to those on Mars, but there are important differences as well.

The most extensive and morphologically diverse chaotic terrains on Mars are geographically restricted to one part of the planet, the circum-Chryse region^8^. However, the planet includes other, much smaller, chaotic terrains, some situated thousands of kilometers apart^8^. This geographic distribution has been interpreted to reflect parts of a global hydrosphere that became unstable and produced the planet’s deepest collapsed terrains^7, 9^. Similarly, on Mercury, the main area of chaotic terrain development is distantly positioned from other smaller occurrences (Fig. S7A). On both planets, these terrains include (1) the presence of vast fields of mesas and knobs that cover regional depressions; (2) the preserved relics of pre-existing landforms (e.g., faults and crater rims) ^4-7^ (Fig. 5C; Fig. S15); (3) characteristic collapse depths of several hundred meters to a few kilometers^4-7^ (Fig. 7; Figs. S3, S4, S15); (4) transitional landscapes reflecting varying degrees and multiple stages of collapse^4-7^ (Fig. 4; Fig. S15). These observations could be reconciled within a theoretical framework in which regional and gradual collapse within global volatile-rich upper crusts produced the chaotic terrains of both Mercury and Mars.

There is, however, a key difference between these chaotic terrains. Martian chaotic terrains, however, commonly involves a passive brittle lid, beneath which volatiles were lost^1-9^, and we do not observe equivalent terrain features on Mercury. On the other hand, the terrains that were the most affected by collapse consist of low-lying plains with sparsely spaced knobs (Fig. 4). Our interpretation is that the knobs within Mercury’s chaotic terrains continued to disaggregate because they were made up of volatile-rich materials, suggesting that during the phase of chaotic terrain development, the volatile-rich upper crustal zone extended to the surface or the near-surface.

Our hypothesis implies that, while different, the environmental conditions of Mars and Mercury permitted the formation, preservation, and controlled loss of volatiles. Perhaps, chaotic terrains in terrestrial planets develop under extreme cold or hot environments that promote the local loss of enormous volumes of buried volatiles.

**
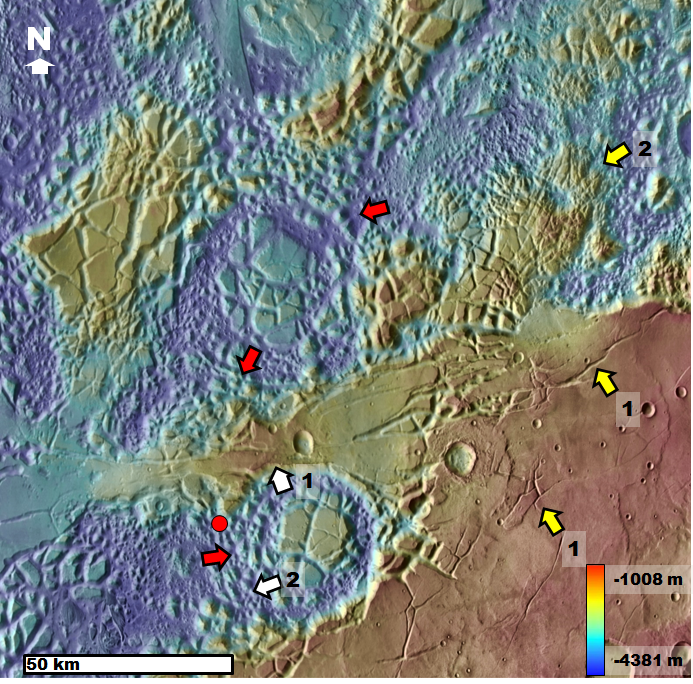
Fig. S15** Martian Chaos includes numerous morphologic attributes, which are strikingly similar to those we have identified in Mercury’s counterparts. For example, the white arrows indicate a section of an impact crater rim, which (1) collapse did not significantly modify, and (2) in which transitions along the crater’s projected perimeter where collapse completely removed the rim (2). Notice that the contact between the two areas is abrupt (red dot). The red arrows identify chaotic terrain-forming promontories, which follow the curvatures of collapsed impact crater margins. The yellow arrow labeled “1” shows highland areas, which extensional faults extensively fracture. The yellow arrow labeled “2” shows chaotic terrain-forming promontories with orientations consistent with those of nearby faults. The image is a combination of THEMIS daytime IR global mosaic and a regional MOLA topographic overlay [THEMIS layer: <http://www.mars.asu.edu/data/>, 100 m/pixel, credit: Christensen, P. R. *et al.* THEMIS Public Data Releases, Image Explorer, Planetary Data System node, Arizona State University, <http://themis-data.asu.edu>, (2006) Date of access: 04/28/2019; MOLA layer: Color-coded shaded-relief MOLA DEM (460 m/pixel, credit: MOLA Science Team, MSS, JPL, NASA)].

**References cited in this section**

1. Carr, M. J. Formation of Martian flood features by release of water from confined aquifers. *J. Geophys. Res*. **84,** 2995–3007 (1979).

2. Baker, V. R. & Milton, D. J. Erosion by catastrophic floods on Mars and Earth. *Icarus* **23,** 27-41 (1974).

3. Rodriguez, J. A. P., Sasaki, S. & Miyamoto, H., Nature and hydrological relevance of the Shalbatana complex underground cavernous system. *Geophysical Research Letters* **30,** 1304, doi:10.1029/2002GL016547 (2003).

4. Rodriguez, J. A. P. *et al.* Outflow channel sources, reactivation, and chaos formation, Xanthe Terra, Mars. *Icarus* **175,** 36-57 (2005).

5. Rodriguez, J. A. P. *et al.* Control of impact crater fracture systems on subsurface hydrology, ground subsidence, and collapse, Mars. *J. Geophys. Res*. **110,** E06003; doi:10.1029/2004JE002365 (2005).

6. Rodriguez, J. A. P. *et al.* Headward growth of chasmata by volatile outbursts, collapse, and drainage: Evidence from Ganges chaos, Mars. *Geophysical Research Letters* **33,** L18203; doi:10.1029/2006GL026275 (2006).

7. Rodriguez, J. A. P. *et al.* Martian outflow channels: How did their source aquifers form, and why did they drain so rapidly? *Scientific Reports* **5,** doi:10.1038/srep13404;(2015).

8. Tanaka, K. L. *et al.* Geologic map of Mars. U.S. Geological Survey Scientific Investigations Map 3292, scale 1:20,000,000,<http://pubs.usgs.gov/sim/3292/> (2014) Date of access: 03/14/2018.42.

9. Clifford, S. M. & Parker, T. J. The evolution of the Martian hydrosphere: Implications for the fate of a primordial ocean and the current state of the northern plains. *Icarus* **154,** 40-79 (2001).

**7. Uncertainties and Future Research Directions**

**7.1. The Origin of the Intercrater Plains: Volcanism within, over, and beneath a Volatile-rich crust?**

The origin of Mercury’s intercrater plains has been attributed to a phase of effusive volcanism^1,2^, which led to the global elimination of a large number of craters ≤100 km in diameter as successive lava flows accumulated ~4.0 to ~4.1 Ga during the Late Heavy Bombardment^3^. However, unlike the Moon, which also exhibits widespread plains, no unequivocal volcanic evidence has been identified on the planet^4^. If the proposed intercrater volcanism occurred over multi-kilometer thick volatile-rich upper crustal materials, the high heat flow could have led to coeval collapse, removing craters and volcanic evidence. High heat flow associated with volcanism in the planet’s intercrater regions could have driven away the vast volumes of upper crustal volatiles, resulting in the deposition of sequences of plains-forming lavas and volatile-free lag deposits. Following the formation of the intercrater plains, ~4.0 to ~4.1 Ga^3^, the planet’s volatile-rich crust became largely stable, probably due to a sharp decrease in global geothermal heat. A consequence of this hypothesis is that this history of collapse on Mercury led to not only chaotic terrain formation, but also a complete upper crustal disaggregation at some locations. An observation that favors this geologic scenario is the fact that our mapping indicates that broad low-lying plains with scattered knobs comprise the chaotic terrain areas, which was most affected by collapse (Fig. 4).

**References cited in this section**

1. Strom, R. G., Trask, N. J., & Guest, J. E. Tectonism and volcanism on Mercury. *Journal of Geophysical Research* **80(17),** 2478–2507 (1975) https://doi.org/10.1029/JB080i017p02478

2. Head, J. W., et al. Volcanism on Mercury: Evidence from the first MESSENGER flyby. *Science* **321(5885),** 69–72 (2008). https://doi.org/10.1126/science.1159256

3. Marchi, S. *et al.* Global resurfacing of Mercury 4.0-4.1 billion years ago by heavy bombardment and volcanism. *Nature* **499,** 59 (2013).

4. Wright, J., Rothery, D. A., Balme, M. R., & Conway, S. J. Constructional volcanic edifices on Mercury: Candidates and hypotheses of formation. *Journal of Geophysical Research: Planets* **123,** 952–971 (2018). https://doi.org/10.1002/2017JE005450

**7.2. The Origin of Extension: Volcanically Induced Inflation?**

The proposed extensional structural patterns mark the intercrater plains (as well as crater rims) (e.g., Fig. 4), and thus, must have developed after or concurrently with their emplacement. The phase of effusive volcanism leading to the emplacement of the intercrater plains occurred during^1^ and possibly as a consequence^2^ of the Late Heavy Bombardment ~4.0 to ~4.1 Ga. Hence, it is conceivable too that the very same phase of volcanism on Mercury that emplaced the intercrater plains also led to surface inflation due to related ascending magma bodies and intrusion of laccoliths beneath a low-density VRC, thereby generating the widespread extensional patterns that later collapsed within the chaotic terrains. Another mechanism that could have led to extension is regional uplifting due to antipodal basin formation^3-6^. Also, it is possible that rates of contraction varied regionally (perhaps due to changes in topography). In this case, the extension could have fractured areas affected by relatively low contraction rates.

**References cited in this section**

1. Marchi, S. *et al.* Global resurfacing of Mercury 4.0-4.1 billion years ago by heavy bombardment and volcanism. *Nature* **499,** 59 (2013).

2. Mojzsis, S. J., Abramov, O., Frank, E. A., & Brasser, R. Thermal effects of late accretion to the crust and mantle of Mercury. *Earth and Planetary Science Letters* **482,** 536–544 (2018). <https://doi.org/10.1016/j.epsl.2017.11.023>

3. Hood L. L. & Artemieva, N. A. Antipodal effects of lunar basin-forming impacts: Initial 3D simulations and comparisons with observations. *Icarus* **193,** 485–502. (2008).

4. Watts, A. W., Greeley, R. & Melosh, H. J. The formation of terrains antipodal to major impacts. *Icarus* **93,** 159-168 (1991).

5. McGovern, P. J., Potter, R. W. K., Collins, G. S., Kring, D. A., Grange, M. L. & Nemchin, A. A. Pulses of magmatic movement triggered by the South Pole-Aitken basin impact, Workshop on Early Solar System Impact Bombardment III, Houston, TX, #3027. (2015). <http://www.lpi.usra.edu/meetings/bombardment2015/pdf/3027.pdf>

6. Kring, D. A., McGovern, P. J., Potter, R. W. K., Collins, G. S., Grange, M. L., & Nemchin, A. A. Was an epoch of lunar magmatism triggered by the South Pole-Aitken basin impact? Workshop on the Early Solar System Impact Bombardment III, Houston, TX, #3009 (2015). <http://www.lpi.usra.edu/meetings/bombardment2015/pdf/3009.pdf.>

**7.3. Global Contraction and the Retention of Extensional Structural Patterns**

Our investigation suggests that the chaotic terrains of Mercury constitute areas of collapse, with a major collapse phase occurring ~1.8 Ga. The chaotic terrain materials exhibit a distribution consistent with structurally controlled collapse. However, these spatial patterns do not necessarily imply active extension during phases of collapse, but rather point to the degassing pathways along older pre-existing zones of crustal weakness. An uncertainty, however, is whether or not the extensional faults would have been sealed due to upper crustal strains during a global contraction phase, which ~3.5 Ga initiated the formation of widespread thrust scarps^1^.

**References cited in this section**

1. Byrne, P. K. *et al.* Widespread effusive volcanism on Mercury likely ended by about 3.5 Ga. *Geophysical Research Letters* **43,** 7408–7416 (2016). https://doi.org/10.1002/2016GL069412
